# Regulation and function of a polarly localized lignin barrier in the exodermis

**DOI:** 10.1038/s41477-024-01864-z

**Published:** 2024-12-02

**Authors:** Concepcion Manzano, Kevin W. Morimoto, Lidor Shaar-Moshe, G. Alex Mason, Alex Cantó-Pastor, Mona Gouran, Damien De Bellis, Robertas Ursache, Kaisa Kajala, Neelima Sinha, Julia Bailey-Serres, Niko Geldner, J. Carlos del Pozo, Siobhan M. Brady

**Affiliations:** 1 Department of Plant Biology and Genome Center, University of California Davis, Davis, CA USA; 2 Centro de Biotecnología y Genómica de Plantas (UPM-INIA/CSIC), Universidad Politécnica de Madrid (UPM)—Instituto Nacional de Investigación y Tecnología Agraria y Alimentaria-CSIC (INIA/CSIC), Campus Montegancedo, Madrid, Spain; 3 Department of Evolutionary and Environmental Biology, Faculty of Natural Sciences, Institute of Evolution, University of Haifa, Haifa, Israel; 4 Department of Plant Molecular Biology, University of Lausanne, Lausanne, Switzerland; 5 Electron Microscopy Facility, University of Lausanne, Lausanne, Switzerland; 6 Centre for Research in Agricultural Genomics (CRAG), CSIC-IRTA-UAB-UB, Barcelona, Spain; 7 Experimental and Computational Plant Development, Institute of Environmental Biology, Utrecht University, Utrecht, the Netherlands; 8 Center for Plant Cell Biology, Department of Botany and Plant Sciences, University of California Riverside, Riverside, CA USA; 9 Department of Plant Biology, University of California Davis, Davis, CA USA; 10 Howard Hughes Medical Institute, University of California Davis, Davis, CA USA

**Keywords:** Cell wall, Cell fate

## Abstract

Multicellular organisms control environmental interactions through specialized barriers in specific cell types. A conserved barrier in plant roots is the endodermal Casparian strip (CS), a ring-like structure made of polymerized lignin that seals the endodermal apoplastic space. Most angiosperms have another root cell type, the exodermis, that is reported to form a barrier. Our understanding of exodermal developmental and molecular regulation and function is limited as this cell type is absent from *Arabidopsis thaliana*. We demonstrate that in tomato (*Solanum lycopersicum*), the exodermis does not form a CS. Instead, it forms a polar lignin cap (PLC) with equivalent barrier function to the endodermal CS but distinct genetic control. Repression of the exodermal PLC in inner cortical layers is conferred by the *SlSCZ* and *SlEXO1* transcription factors, and these two factors genetically interact to control its polar deposition. Several target genes that act downstream of *SlSCZ* and *SlEXO1* in the exodermis are identified. Although the exodermis and endodermis produce barriers that restrict mineral ion uptake, the exodermal PLC is unable to fully compensate for the lack of a CS. The presence of distinct lignin structures acting as apoplastic barriers has exciting implications for a root’s response to abiotic and biotic stimuli.

## Abstract (web summary)

In tomato roots, the exodermis forms a genetically distinct polar lignin cap (PLC) barrier from the Casparian strip. SlSCZ and SlEXO1 repress PLC deposition in inner layers. The PLC cannot fully compensate for the CS as a mineral ion barrier.

## Main

The control of external mineral ions and water entry by roots is essential for plant survival. In vascular plants, the endodermis is the innermost root cell layer that controls their passive diffusion through the cell wall (apoplastic) space by the formation of a barrier called the Casparian strip (CS)^1^. The molecular pathway controlling endodermis specification and differentiation has been well elucidated in *Arabidopsis thaliana*^2–7^. The non-cell autonomous SHORT-ROOT (SHR) transcription factor specifies endodermal identity^8^. Its subsequent transcriptional cascade coordinates the expression of factors that synthesize, deposit and position the CS^8–11^. The CS is composed of polymerized lignin which impregnates the endodermal primary cell wall in a precisely localized ring around its central axis^12,13^. Hallmarks of this positioning include tight membrane adhesion and the action and localization of protein domains comprising CASPARIAN STRIP DOMAIN PROTEIN (CASP)/CASP-like and the ENHANCED SUBERIN 1 (ESB1) proteins^2–5,14^. The transcription factor *AtMYB36* positively regulates the expression of the *AtCASP1*, *AtPER64* and *AtESB1* genes that are necessary to define CS positioning as well as its polymerization. Mutation of *AtMYB36* (*atmyb36*) results in an absent CS^15,16^, ectopic lignin deposition in endodermal cell corners as well as disruption of CS barrier function^15,17^. The *SCHENGEN3/SCHENGEN1/CASPARIAN STRIP INTEGRITY FACTOR2(SGN3/SGN1/CIF2)* pathway acts as an elegant surveillance system to perceive defects in CS integrity. If such defects occur, this pathway activates compensatory lignification and suberization^6,7^. Both the *atmyb36* and the *atmyb36atsgn3* mutants have a drastic perturbation of ion homoeostasis and growth^15,17^, proving the important function of the endodermal CS in whole plant growth. After CS deposition, suberin lamellae eventually coat the entire endodermal cell wall surface in a second differentiation stage^18,19^.

Many plant species, but not *Arabidopsis*, contain an additional cell type with barrier function, the exodermis or the hypodermis, herein referred to as the exodermis. Located underneath the epidermis, the exodermis is present in more than 90% of 200 angiosperms previously examined^20–22^. On the basis of staining technology available at that time, it was shown that exodermal cell walls contain lignin- or suberin-related compounds in a variety of deposition patterns, many of which included a broad band along the radial and anticlinal cell walls. At that time, a CS was thought to be composed of both suberin and lignin and the authors therefore concluded that the exodermis in these species possess a CS. More recently, however, a CS has been conclusively demonstrated to be composed solely of polymerized lignin, which is necessary for its function as an apoplastic barrier^2^. Unanswered questions include whether the exodermis contains lignin or suberin, its precise subcellular location and which genes control exodermal differentiation.

We previously demonstrated that the tomato (*Solanum lycopersicum*, cv. M82) exodermis is both lignified and suberized in two differentiation steps^23,24^. First, a polar lignin cap (PLC) is deposited and localized on the epidermal face of exodermal cells, followed by suberization of the entire cell surface. Here we demonstrate that in tomato, the exodermal lignin barrier is not a CS, but the PLC nevertheless functions as an apoplastic diffusion barrier. We further demonstrate that while regulation of endodermal CS differentiation is generally conserved between *Arabidopsis* and tomato, the regulatory pathway for the PLC in exodermal differentiation is genetically distinct. Two transcription factors, *SlEXO1* and *SlSCHIZORIZA (SlSCZ)*, restrict the deposition of the PLC and suberin to the exodermis and genetically control the polarity of the PLC. Phenotypic analysis of these mutants shows that the exodermal PLC is unable to fully functionally compensate for the lack of the endodermal CS, and that it has similar and unique properties compared with the endodermal CS. Both transcription factors coordinate cell wall and lignin-related pathways associated with exodermal-enriched factors whose function is associated with the PLC. Our findings reveal genetically distinct pathways for the production of multiple cell wall barriers that control how a root interacts with the environment.

## Results

### The exodermal polar lignin cap is an apoplastic barrier

**Fig. 1:**
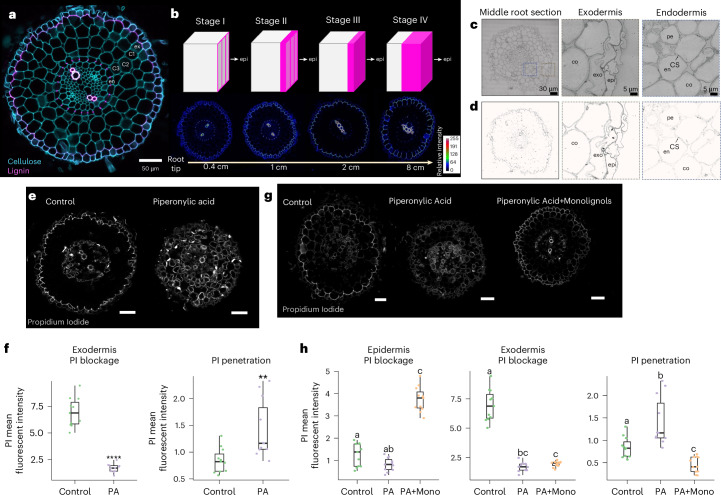
Exodermal lignin is polarized and serves as an apoplastic barrier. **a**, Tomato root cross-section stained with basic fuchsin (pink) and calcofluor white (blue) for lignin and cellulose, respectively. ex, exodermis; C1, cortex 1; C2, cortex 2; C3, cortex 3; en, endodermis. Scale bar, 50 µM. **b**, Model for exodermis lignin deposition. Top: each cube represents an exodermis cell. The epidermis (epi) face is on the right. Pink represents lignin. Bottom: root cross-sections from 0.4 cm, 1 cm, 2 cm and 8 cm from the root tip, stained with basic fuchsin. **c**, TEM of a middle root section stained with potassium permanganate. Left: root section. Middle: magnified exodermis (grey dotted square). Right: magnified endodermis (blue dotted square). co, cortex; pe, pericycle. Identical results were observed in 3 independent experiments. **d**, Same images as in **c** with adjusted contrast to highlight lignin. Dark arrows highlight lignin deposition in the exodermis cell wall. **e**, Tomato root sections from control and PA-treated plants for 24 h and next incubated with the apoplastic tracer PI for 30 min. Scale bars, 50 µM. **f**, Quantification of PI blockage at the exodermal PLC and penetration into cortex cells in control and PA-treated plants (*n* = 11). PI blockage ****P = 9.5 × 10^−11^; PI penetration **P = 0.0046. *statistical significance (one-way ANOVA). Boxplots show the median, 25th−75th percentiles (interquartile range (IQR)), and minima and maxima (whiskers). **g**, Tomato root cross-sections from control, PA-treated and PA+monolignol-treated plants, followed by incubation with PI. Scale bars, 50 µM **h**, Quantification of PI blockage at the epidermis, exodermal PLC and cortex cell penetration in control and PA-treated plants and PA+monolignol plants, followed by incubation with PI (*n* = 11). Statistical significance was determined using one-way ANOVA with a post hoc Tukey HSD test; *P* < 0.05. Letters indicate statistically different groups. Boxplot definitions are as in **f**.

**Extended Data Fig. 1:**
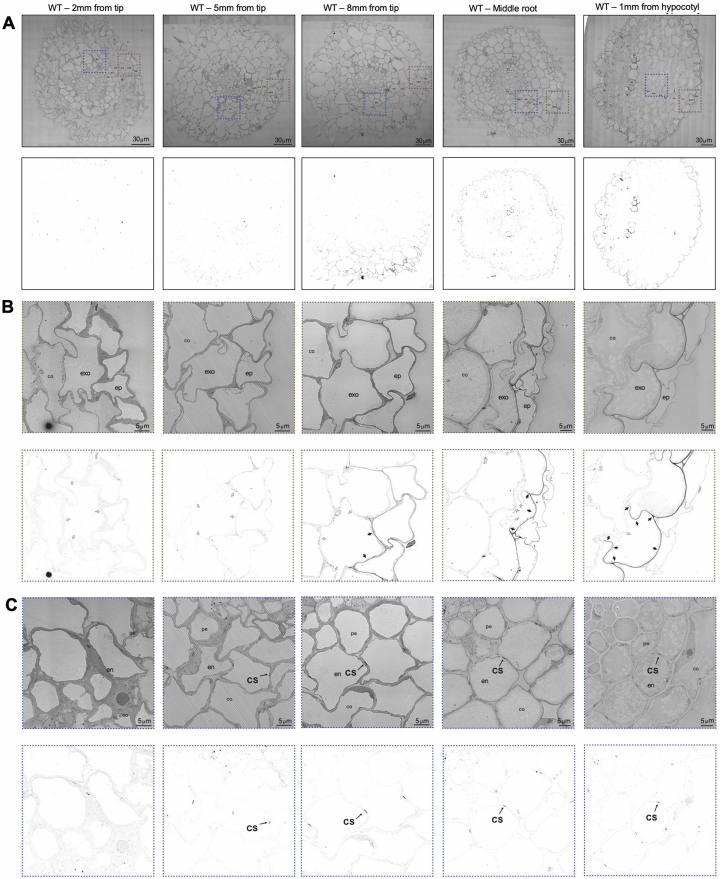
Ultrastructural lignin deposition in the exodermis and endodermis. **a**. TEM panoramas from wild-type root cross-sections at 2, 5, and 8 mm from the tip, a middle section of the root, and at 1mm from hypocotyl stained with KMn0_4_. Dark deposits indicate electron dense Mn0_2_ precipitation caused by reaction with lignin. Lower panels show the same pictures with an adapted contrast that highlights the dark deposits. co = cortex, exo = exodermis, ep = epidermis, en = endodermis, pe =pericycle, scale bars=30 µm. **b**. Close-up of the exodermis area from panels in A (zone defined with brown dashed lines). Lower panels show the same pictures with an adapted contrast that highlights the dark deposits. Dark arrows highlight lignin deposition in the exodermis cell wall. White arrows highlight the absence of lignin in the exodermis cell wall. Lignin deposition begins at 8mm from the root tip and is oriented toward the epidermis. co = cortex, exo = exodermis, ep = epidermis, scale bars=5 µm. **c**. Close-up of the endodermis area from panels in A (zone defined with blue dashed lines). Lower panels show the same pictures with an adapted contrast that highlights the dark deposits. The CS is fully lignified at 5mm from the root tip. co = cortex, en = endodermis, pe = pericycle, cs = Casparian strip, scale bars=5 µm.

**Extended Data Fig. 2:**
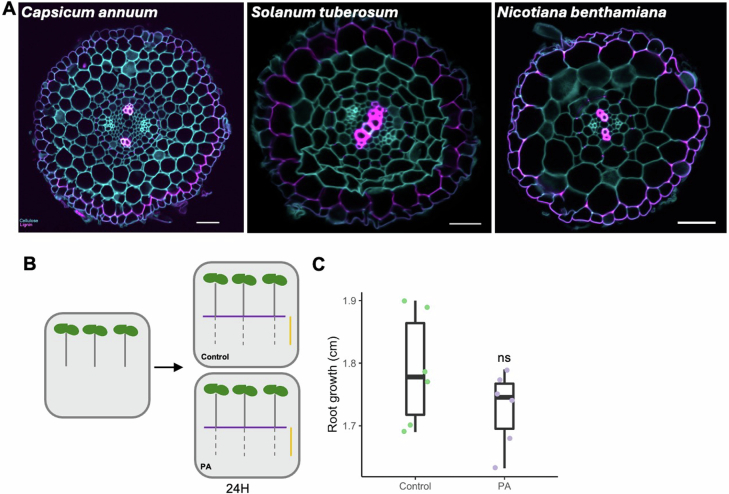
The PLC is conserved in the Solanaceae family and piperonylic acid does not affect root growth. **a**. Cross sections of Solanum species and *Nicotiana benthamiana* stained with basic fuchsin and calcofluor. Left panel: *Capsicum annuum* variety Alliance bell pepper. Middle panel: *Solanum tuberosum* group Andigenum. Right panel: *Nicotiana benthamiana*. Scale bar = 50 µM. **b**. Schematic representation of the experimental design to test the PA. 4-day-old plants after germination were transferred to MS media + 1% sucrose with and without 200 µM of PA. After 24 hours, root growth was measured for the part of the root that grew after transferring. Orange horizontal bar = newly grown root after transferring. Scale Bar = 50 µm **c**. Root growth after 24H in control and PA treatment for the new root zone after transferring n=6. Statistical significance test determined by one-way ANOVA p-value <0,05. Box plots show median, IQR (25^th^−75^th^ percentile), and whiskers represent minima and maxima. Error bars s.d.

The temporal nature of exodermal PLC deposition was characterized in 5-day-old tomato (*S. lycopersicum*, cv. M82) seedlings using histochemical staining with basic fuchsin (lignin) and calcofluor white (cellulose) to counterstain the cell walls (Fig. 1a,b). These data were complemented with transmission electron microscopy (TEM) combined with potassium permanganate staining to visualize electron-dense polymerized lignin (Fig. 1c,d and Extended Data Fig. 1)^25^. Exodermal lignification occurs after endodermal CS deposition in the early maturation zone. Lignin is first deposited at exodermal–epidermal cell corners, at ~0.4 cm from the root tip which we term Stage 1 of exodermal lignification (Fig. 1b–d). In Stage 2 of exodermal differentiation, ~1 cm from the root tip, lignin increases at exodermal cell corners and becomes deposited along the entire epidermal face of exodermal cells (Fig. 1b). Stage 3 of exodermal differentiation occurs ~2 cm from the root tip, with lignin levels increasing and spreading along the anticlinal cell walls towards the first inner cortical cell layer. This stage is maintained from 2 to 8 cm from the root tip, near the root–hypocotyl junction where Stage 4 of exodermal differentiation comprises maximal lignification of PLC with ~2/3 of the anticlinal cell wall (Fig. 1b). Polymerized lignin levels additionally increase at the epidermal face (Fig. 1b). This exodermal PLC is conserved in all solanaceous species examined (Extended Data Fig. 2a).

The formation of an apoplastic barrier is a hallmark of CS function. To test whether the exodermal PLC has an equivalent function, we used propidium iodide (PI) as an apoplastic tracer^12,26^. PI apoplastic transport was still blocked by the exodermal cap after 30 min of incubation as indicated by the lack of PI penetrance to inner cortical cell layers (Fig. 1e,f). Vascular PI presence was due to PI absorption within the meristem and led to subsequent transport through the xylem. Piperonylic acid (PA), a monolignol biosynthetic inhibitor, was used to further demonstrate that exodermal PLC barrier function is dependent upon lignin biosynthesis^12,27^. Exposure to 200 µM PA for 24 h does not interfere with root growth but does disrupt root lignin biosynthesis within the endodermis and exodermis (Extended Data Fig. 2b,c). In PA-treated plants, PI staining of cortical cells was observed after exposure (Fig. 1e). In addition, the PI signal in the exodermal PLC was more intense in control relative to PA-treated plants (Fig. 1f). The dependence of exodermal PLC barrier function on lignin biosynthesis was further determined by complementation of the PA inhibitor-induced defects by the addition of two components of angiosperm lignin, the monolignols coniferyl- and sinapyl-alcohol (20 µM each), as previously used to demonstrate CS barrier function in *Arabidopsis* endodermis^12^. Treatment with both PA and monolignols restored lignin levels in the exodermis, increased lignin levels within the epidermis and blocked PI transport at the epidermis (Fig. 1g,h).

### Exodermal and endodermal differentiation are genetically distinct

**Fig. 2:**
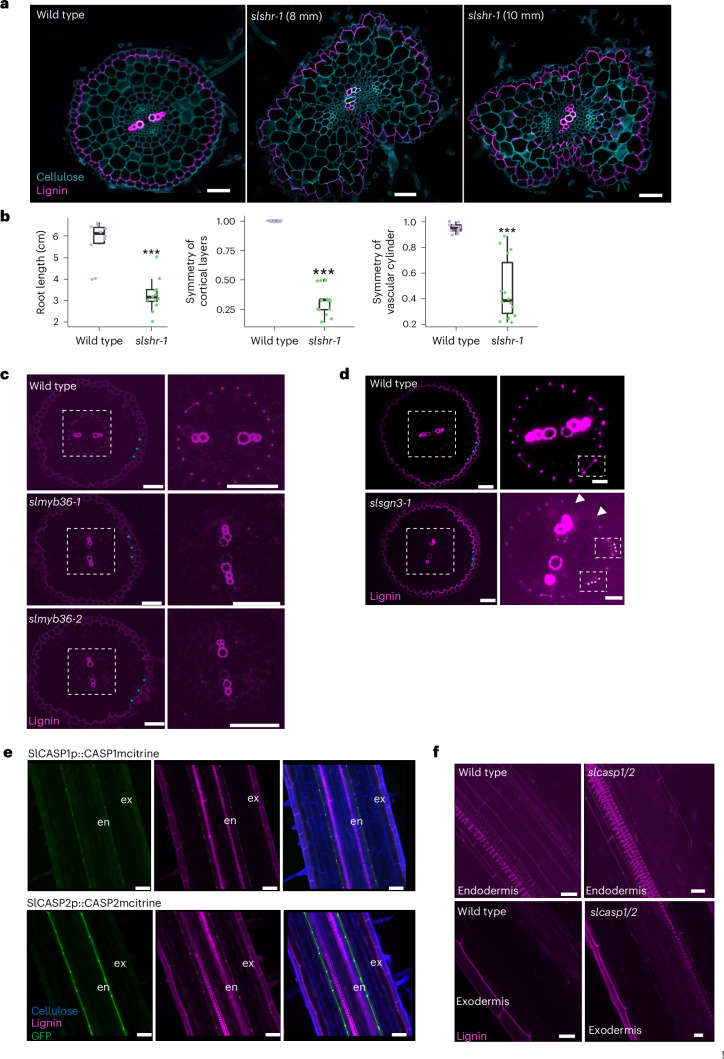
Known endodermal developmental regulators do not control exodermal differentiation. **a**, Root sections of 4-day-old plants stained with basic fuchsin (pink) and calcofluor white (blue) for lignin and cellulose. Left: the wild-type endodermis has a CS and the exodermis a polar lignin cap (PLC). Middle: in the *slshr-1* mutant, the CS is absent earlier (8 mm from the tip). Right: ectopic lignin deposition is observed in cells that surround the vasculature (10 mm from the tip). The exodermal PLC is normal. **b**, Left: root length of 4-day-old wild type and *slshr-1* (*n* = 12). Middle: cortical symmetry (exodermis included) calculated as the minimum/maximum number of cortex layers in radial cross-section (*n* = 10) in wild type and *slshr-1*. Right: vascular cylinder symmetry measured by minimum/maximum distance across its centre (*n* = 10). Statistical tests with one-way ANOVA; ***P < 0.005. Boxplots show the median, 25th−75th percentiles (IQR), and minima and maxima (whiskers). **c**, Two independent mutant alleles of *SlMYB36* (*slmyb36-1* and *slmyb36-2*) lack an endodermal CS but retain a wild-type exodermal PLC. Left: root cross-section stained with basic fuchsin, blue asterisks show a normal exodermal PLC. Right: magnified view of vascular cylinder and endodermis. Results were consistent across 3 experiments. **d**, *SlSGN3* mutation (*slsgn3-1*) leads to an interrupted, non-continuous CS. Left: root section stained with basic fuchsin, blue asterisks show a normal exodermal PLC. Right: magnified view of vascular cylinder and endodermis. Small square, top views showing wild-type versus interrupted CS in *slsgn3-1* (white asterisks and triangles). Brightness was adjusted in the magnified pictures for clarity. Results were consistent across 3 experiments. **e**, Translational fusions of SlCASP2 and SlCASP1 with mCitrine under their respective promoters localize specifically in the endodermis, not the exodermis. Cell walls are stained with calcofluor white (blue), lignin with basic fuchsin (pink), and mCitrine is visualized in the GFP channel (green). Results were consistent across 6 experiments. **f**, *slcasp1/2* double mutant did not affect endodermal CS or exodermal PLC formation relative to wild type. Lignin is stained with basic fuchsin (pink). Results were consistent across 4 experiments. All CRISPR or reporter lines were generated via *A. tumefaciens* transformation unless otherwise noted. All scale bars, 50 µm.

**Extended Data Fig. 3:**
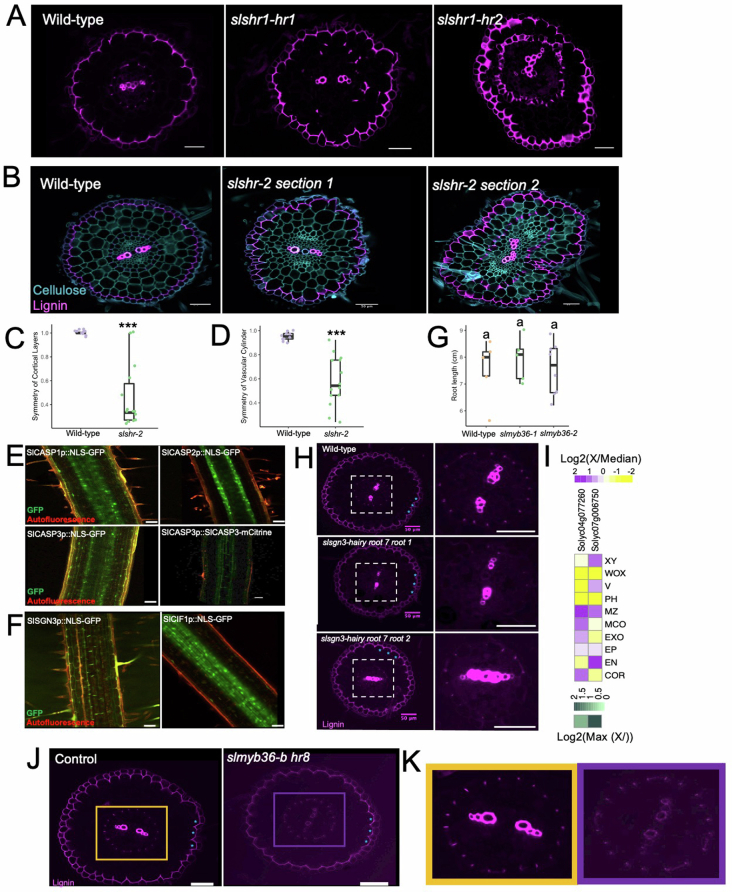
Endodermal regulators do not regulate exodermal differentiation. **a**. Hairy root cross-sections stained with fuchsin of wild-type (transformed with *R.rhizogenes* with no binary plasmid) and *shr-1* mutants from 2 independent hairy root lines. Left panel = wild-type, middle panel = *slshr1 hairy root Line 1 (slshr1-hr1)*, right panel = *slshr1 hairy root line 2 (shr1-hr2)*. Scale Bar = 50 µm **b**. Cross sections of wild-type and the *shr-2* mutant allele (*A. tumefaciens*-transformed) from two different parts of the root - 8 mm from the tip (middle) and 10 mm from the tip (right). The mutant layer undergoes ectopic lignin deposition in more mature regions of the root. Scale Bar = 50 µm **c**. Left panel: Symmetry of cortical layers (including the exodermis). This is calculated as the minimum number of cortex layers observed divided by the maximum number of cortex layers in wild type and *shr-2* mutant roots (p-value=2.86e06, *=statistical significance as determined by one-way ANOVA n=14. Box plots show the median, error bars s.d. IQR (25^th^−75^th^ percentile) and whiskers represent minima and maxima. **d**. Symmetry of the vascular cylinder was calculated as the minimum distance across the centre of the vascular cylinder divided by the maximum distance across the centre of the vascular cylinder (p-value=1.36e05, *=statistical significance as determined by one-way ANOVA n=14). Box plots show the median, IQR (25^th^−75^th^ percentile) and whiskers represent minima and maxima. **e**. Hairy roots with transcriptional fusions for *CASP1-3* genes to GFP and a translational fusion of *SlCASP3* to mCitrine transformed. Scale Bar = 50 µm **f**. Hairy roots with *SlSGN3* and *SlCIF2* transcriptional fusion to GFP. Scale Bar = 50 µm. **g**. Root length in cm of two *slmyb36* independent alleles. Statistical significance as determined by one-way ANOVA with a post-hoc Tukey HSD test; pvalue<0.05 n=5. Box plots show the median,IQR (25^th^−75^th^ percentile) and whiskers represent minima and maxima. **h**. Mutation of *SlSGN3* in the hairy root line 7 results in an absent CS, or in a perturbed CS in the radial axis. Left panels = whole root section stained with fuchsin. Blue asterisks show the polar lignin cap in the exodermis is not affected.; right panels = magnified image of vascular cylinder and endodermis layer. Scale Bar = 50 µm. Results were consistent across three experiments. **i**. Expression patterns of two *SlMYB36* homologs from the *MYB36* phylogeny in individual cell-types from data in Kajala et al. ^23^. Legend on the left represents the Log2 maximum value across all conditions and legend on the right represent the Log2(x)/Median. **j**. Cross section of *R. rhizogenes* generated mutant allele *myb36-b hairy root line 8* (*slmyb36-b hr-8*) for *Solyc04g077260*. Whole root cross section. Blue asterisks show the polar lignin cap in the exodermis is not affected. **k**. Increased magnification of the endodermis (From panel J – see corresponding orange and purple boxes). Results were consistent across three experiments Scale bar = 50 µm.

Although structurally distinct, the tomato exodermis forms an apoplastic barrier, the PLC, with a function similar to that of endodermal CS. Therefore, one working hypothesis is that genes that regulate endodermal differentiation also regulate exodermal differentiation. Alternatively, a distinct set of genes regulates differentiation of these cell types. Recent evidence in maize demonstrates that mutations in two of the three *ZmSHR* paralogues reduce cortex layer number and probably cortex specialization^28^. The tomato exodermal layer results from asymmetric divisions of the cortex–endodermal initial at the stem cell niche^29^. We therefore tested whether *SlSHR* regulates formation of the exodermal PLC. Both *Rhizobium rhizogenes* and *Agrobacterium tumefaciens*-generated *SlSHR* CRISPR-Cas9 mutant alleles have defects in endodermal specification and differentiation, including the absence of the presumed endodermal layer due to loss of the CS in early developmental stages (Fig. 2a and Extended Data Fig. 3a,b). In more mature areas of the root, aberrant CS formations due to disorganized cell divisions are observed (Fig. 2a). Both *slshr-1* and *slshr-2* alleles additionally have asymmetric radial patterning in the ground tissue layers and the vascular cylinder and short roots (Fig. 2b and Extended Data Fig. 3c,d). These phenotypes demonstrate that *SlSHR* function is largely conserved between *Arabidopsis* and tomato for endodermal patterning and differentiation. A deviation from the *Arabidopsis* mutant phenotype is the radial asymmetry in the cortex cell layers (including the exodermis) and aberrant lignin deposition in the mutant layer (Fig. 2a,b). However, all *slshr* alleles had a wild-type exodermal lignin cap (Fig. 2a).

Loss of function *atmyb36* mutants lack the CS and present ectopic lignification in endodermal cells^15,17^. Phylogenetic analysis revealed two possible tomato MYB36 homologues of *Arabidopsis* *AtMYB36 (At5g57620)*: *Solyc07g006750* and *Solyc04g077260* (Supplementary Fig. 1a). *Solyc07g006750* is more phylogenetically related to *AtMYB36* (Supplementary Fig. 1b) and its ribosome-associated transcript abundance is enriched in the endodermis (Extended Data Fig. 3i)^23^. *Solyc04g077260* is a recently reported homologue of *AtMYB36* (ref. ^30^) (Supplementary Fig. 1a). CRISPR/Cas9 hairy root mutants of *Solyc07g006750* (herein *SlMYB36*) but not *Solyc04g077260* (herein *SlMYB36-b*) lack a CS (Extended Data Fig. 3j,k). *A. tumefaciens*-transformed CRISPR/Cas9-edited *slmyb36* mutant alleles lack a CS but have no changes in ground tissue radial patterning and root growth (Fig. 2c and Extended Data Fig. 3g). In contrast to *atmyb36* mutants^15,17^, *slmyb36* mutants have minimal ectopic lignification within the endodermis. Neither *slmyb36* nor *slmyb36-b* mutants showed defects in the exodermal PLC (Fig. 2c and Extended Data Fig. 3j). These results are consistent with a conserved function for *SlMYB36* in endodermal differentiation, but not in exodermal differentiation.

AtCASP proteins act as a scaffold to guide lignin biosynthesis enzymes to the CS domain^3^. We identified four putative tomato AtCASP homologues (Solyc02g088160, Solyc06g074230, Solyc09g010200 and Solyc10g083250) (Supplementary Fig. 2). Three of these have the extracellular loop1 (EL1) protein domain that is conserved in spermatophytes (Solyc06g074230, Solyc09g010200 and Solyc10g083250)^31^ and were chosen for further study. Transcriptional GFP reporters demonstrated that *SlCASP1 (Solyc06g074230)* is expressed primarily in the endodermis with some expression in inner cortical and exodermal cells, *SlCASP2 (Solyc10g083250)* is primarily expressed in the endodermis and *SlCASP3 (Solyc09g010200)* is expressed in the endodermis, cortex and exodermis (Extended Data Fig. 3e). SlCASP1 and SlCASP2, but not SlCASP3 translational mCitrine reporters localize to the endodermal CS domain and not at the exodermal PLC domain (Fig. 2e and Extended Data Fig. 3e). *SlCASP1* and *SlCASP2* gene (*slcasp1slcasp2*) mutant alleles showed no phenotype in the endodermal CS or the exodermal PLC (Fig. 2f), suggesting a high degree of functional redundancy among CASPs, similar to what is observed in *Arabidopsis*^32^.

The AtSGN3 receptor kinase along with the AtCIF peptide controls the CS integrity surveillance system^6,7^. Phylogenetic analyses revealed one close homologue to *AtSGN3*/*At4g20140*, the *Solyc05g007230* gene, and one to the *AtCIF1*/*At2g16385* and *AtCIF2*/*At4g34300* genes, *Solyc01g109900* (also previously annotated as *Solyc01g099895*) (Supplementary Fig. 3). Transcriptional GFP reporters for *SlSGN3* and *SlCIF1* demonstrated a *SlSGN3* expression domain in the endodermis, inner cortex and exodermis, while the *SlCIF1* expression domain is in the vasculature as in *Arabidopsis*^6,7^ (Extended Data Fig. 3f). CRISPR/Cas9 mutant alleles revealed an interrupted CS as observed for *Arabidopsis sgn3* (Fig. 2d and Extended Data Fig. 3h)^33^. The exodermal PLC is not affected in these mutants (Fig. 2d and Extended Data Fig. 3h). These data collectively demonstrate that *SlSHR*, *SlMYB36*, *SlCASP1*, *SlCASP2* and *SlSGN3* are orthologues of *Arabidopsis* endodermal CS regulators, that endodermal differentiation is conserved between *Arabidopsis* and tomato, and that tomato exodermal PLC differentiation is genetically distinct from endodermal differentiation.

### Transcriptional repressors of exodermal differentiation

**Fig. 3:**
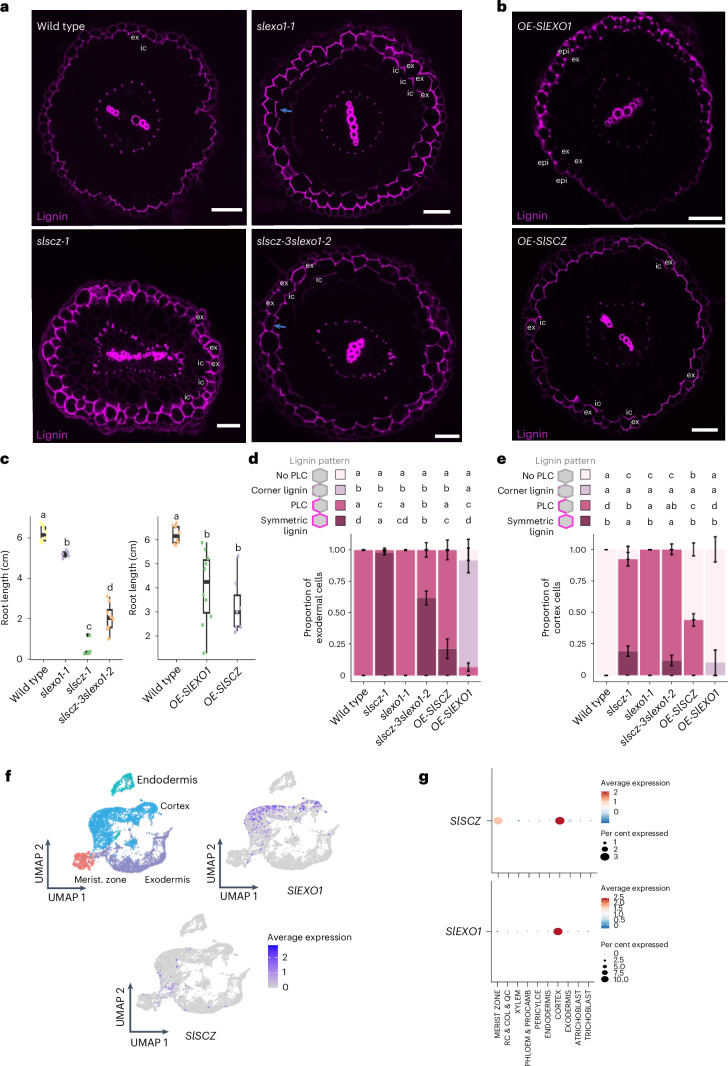
*SlEXO1* and *SlSCZ* repress lignification in the inner cortical layer(s). **a**, The *slexo1-1* mutant shows an additional PLC in the first inner cortical layer, while the *slscz-1* mutant shows additional PLC and occasional full lignification in cortex layers and non-polar lignification in the exodermis. The first inner cortex layer contains a PLC. The *slscz-3* *slexo1-2* double mutant shows reduced symmetric lignification compared with *slscz-1*. **b**, Overexpression of *SlEXO1* (*OE-SlEXO1*) reduces exodermal PLC and increases epidermal lignin. Overexpression of *SlSCZ* (*OE-SlSCZ*) causes ectopic lignification in some inner cortex cells. ic, inner cortex. Pink, lignin. **c**, Root length of wild type, the *slexol-1*, *slscz-1* and *slscz-3slexol-2* mutants, and *SlEXO1* and *SlSCZ* overexpressors (wildtype, *n* = 9; *slscz-1*, *n* = 6; *slexo1*, *n* = 5; *OE-SlEXO1*, *n* = 10; *OE-SlSCZ*, *n* = 7). Significance was determined using one-way ANOVA with post hoc Tukey HSD test; *P* < 0.05. Boxplots show median, 25th−75th percentiles (IQR), and minima and maxima (whiskers). **d**, Proportion of exodermal cells showing no PLC, corner lignin, the PLC and fully (symmetric) lignin in wild type, *slscz-1*, *slexo1-1*, *slscz-3* *slexo1-2*, *OE-SlSCZ* and *OE-SlEXO1* (wild type, *n* = 8; *slscz-1*, *n* = 8; *slexo1,* *n* = 6; *OE-SlEXO1*, *n* = 10; *OE*-*SlSCZ*, *n* = 12). Lignin (pink) patterns are represented in hexagons. Significance was determined using one-way ANOVA with a post hoc Tukey HSD test (*P* < 0.05). Error bars denote s.d. **e**, Proportion of inner cortex cells with no PLC, corner lignin, PLC and fully (symmetric) lignification in wild type, *slscz-1, slexo1-1, slscz-3slexo1-2*, *OE-SlSCZ* and *OE-SlEXO1* (wild type, *n* = 8; *slscz-1*, *n* = 8; *slexo1*, *n* = 6; *OE-SlEXO1*, *n* = 10; *OE*-*SlSCZ*, *n* = 12). Lignin patterns are represented in hexagons. Significance was determined using one-way ANOVA with a post hoc Tukey HSD test (*P* < 0.05). Error bars denote s.d. **f**, Uniform manifold approximation and projection (UMAP) of cortex/endodermis/exodermis cells re-embedded from the general projection (Extended Data Fig. 8e). *SlEXO1* is expressed in the cortex, and *SlSCZ* is expressed in the meristem, cortex and exodermis. Colour scale shows log_2_-normalized unique molecular identifier counts. **g**, Cell type-specific expression profiles for *SlEXO1* and *SlSCZ*. Dot size indicates the percentage of expressing cells and colours represent scaled average expression across developmental stages, with warmer colours indicating higher expression levels. RC, root cap; QC, quiescent centre; Col, columella; Procamb, procambium. All scale bars, 50 µm.

**Extended Data Fig. 4:**
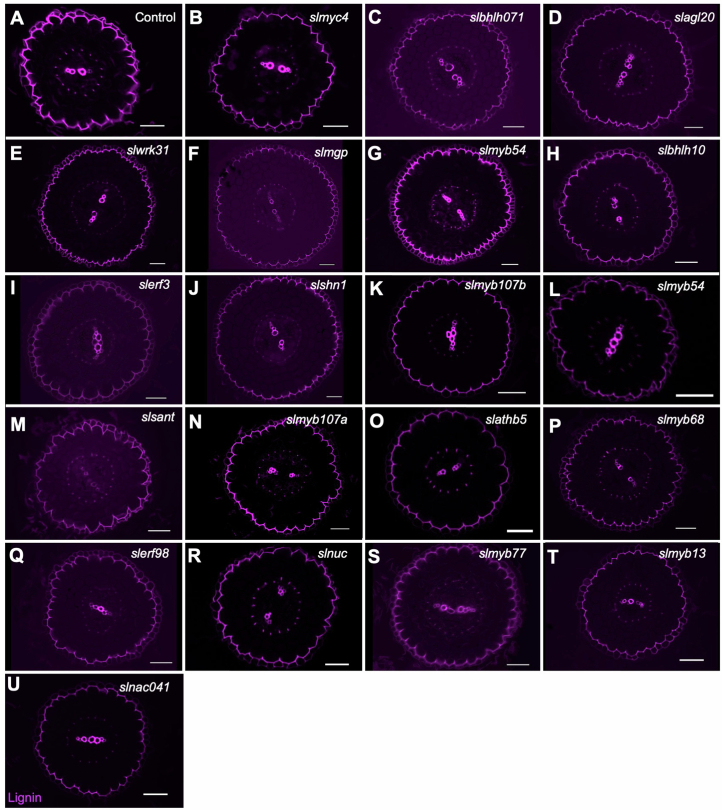
CRISPR-edited hairy root mutants for exodermal-enriched transcription factors. Guide RNAs and edited mutations are found in Supplemental Table 1. For all panels: Root cross sections stained with Basic Fuchsin. **a**. Control = non-transformed hairy root. **b**. *slmyc4* hairy root line 2 (*Solyc08g005050)*. **c**. *slbhlh071-1* hairy root line 9 (*Solyc11g010340)*. **d**. *slagl20* hairy root line 9 (*Solyc01g093965)*. **e**. *slwrk31* hairy root line 3 *(Solyc05g007110)*. **f**. *slmgp* hairy root line 17 (*Solyc01g099340)*. **g**. *slmyb54* hairy root line 2 (*Solyc10g081320)*. **h**. *slblh10* hairy root line 16A (*Solyc06g074120)*. **i**. *slerf3* hairy root line 3 *(Solyc06g082590)*. **j**. *slshn1 hairy root line 5* (*Solyc01g005630)*. **k**. *slmyb107b* hairy root line 6 *(*Solyc02g088190). **l**. *slmyb54-1* (*Solyc03g093890)*. **m**. *slsant* hairy root line 7 (*Solyc10g080960)*. **n**. *slmyb107a* hairy root line 15 (*Solyc02g079280)*. **o**. *slathb5* hairy root line 8 (*Solyc01g096320)*. **p**. *slmyb68* hairy root line 7 *(Solyc11g069030)*. **q**. *slerf98* hairy root line 12 (*Solyc05g050790)*. **r**. *slnuc* hairy root line 11 (*Solyc09g074780)*. **s**. *slmyb77* hairy root line 6 (*Solyc04g079360)*. **t**. *slmyb13* hairy root line 7 (*Solyc08g008480)*. **u**. *slnac041* hairy root line 8 (*Solyc01g009860)*. All scale bars = 50 µm.

**Extended Data Fig. 5:**
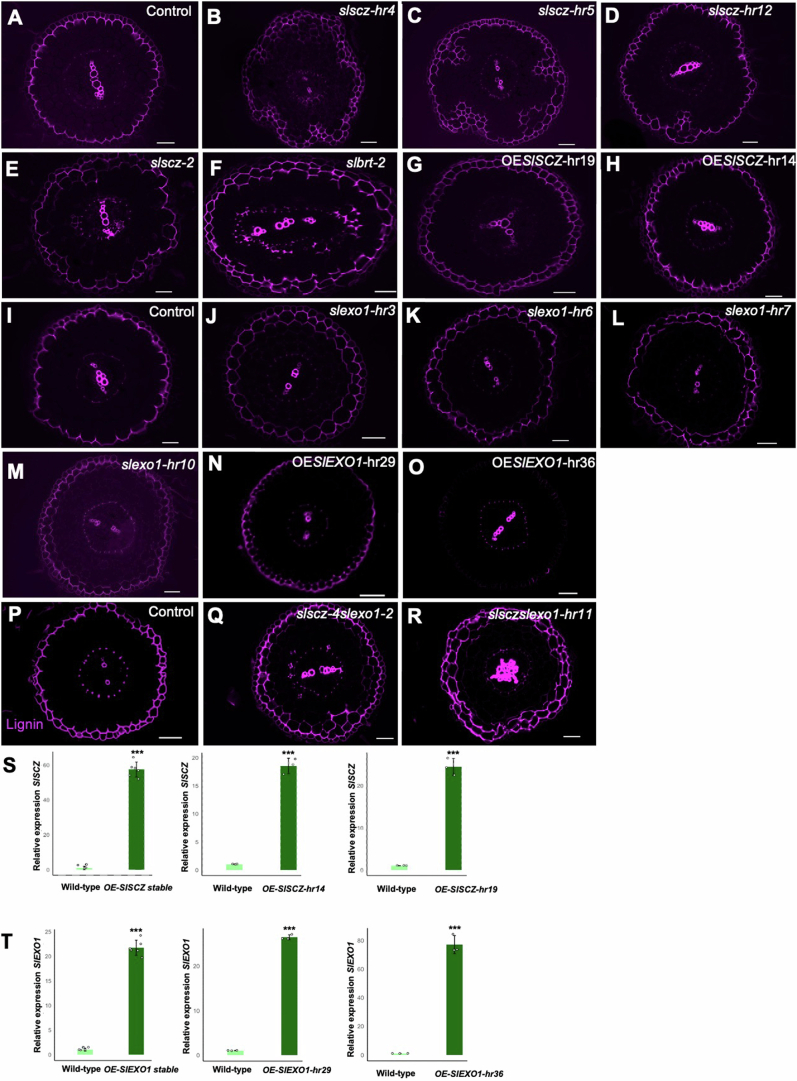
*slscz* and *slexo1* overexpressor and mutant lines. **a**. Control cross section stained with fuchsin. **b**. *slscz hairy root line 4* (*slscz-hr4*) cross section stained with fuchsin. **c**. *slscz hairy root line 5* (*slscz-hr5*) cross section stained with fuchsin. **d**. *slscz hairy root line 12* (*slscz-hr12*) cross section stained with fuchsin. **e**. *A. tumefaciens*-transformed *slscz-2* line cross section stained with fuchsin. **f**. *slbrt-2* mutant (Kevei et al., ^39^). **g**. *OE-SlSCZ hairy root line 19* (*OE-SlSCZ-hr19*) cross section stained with fuchsin. **h**. *OE-SlSCZ hairy root line 14* (*OE-SlSCZ-hr14*) cross section stained with fuchsin. **i**. Control cross section stained with fuchsin. **j**. *slexo1 hairy root line 3* (*slexo1-hr3*) cross section stained with fuchsin. **k**. *slexo1 hairy root line 6* (*slexo1-hr6*) cross section stained with fuchsin. **l**. *slexo1 hairy root line 7* (*slexo1-hr7*) cross section stained with fuchsin. **m**. *slexo1 hairy root line 10* (*slexo1-hr10*) cross section stained with fuchsin. **n**. *OE-SlEXO1 hairy root line 29* (*OE-SlEXO1-hr29*) cross section stained with fuchsin. **o**. *OE-SlEXO1 hairy root line 36* (*OE-SlEXO1-hr36*) cross section stained with fuchsin. **p**. Control cross section stained with fuchsin. **q**. *A. tumefaciens*-transformed *slscz-4slexo1-2* double mutant line cross section stained with fuchsin. **r**. *slsczslexo1 hairy root line 11* (*slsczslexo1-hr11*) double mutant cross section stained with fuchsin. All scale bars = 50 µm. **s**. Left panel: Relative expression of *SlSCZ* in OE-SlSCZ stable line and wild type (n= 6) (p-value=3.12e-11, one-way ANOVA). Middle panel: Relative expression of *SlSCZ* in OE-SlSCZ hairy root line 14 and wild type (n= 6) (Hairy root line with no vector) (p-value= 2.47e-05, one-way ANOVA). Right panel: Relative expression levels in OE-SlSCZ hairy root line 19 and wild type (Hairy root line with no vector) (n= 6) (p-value= 3.62e-05, one-way ANOVA) (Signif. codes: 0 ‘***’ 0.001 ‘**’ 0.01 ‘*’ 0.05). Error bars denote s.d. **t**. Left panel: Relative expression of *SlEXO1* in OE-SlEXO1 stable line and wild type (n= 6) (p-value= 1.89e-11, one-way ANOVA). Middle panel: Relative expression of *SlEXO1* in OE-SlEXO1 hairy root line 29 and wild type (Hairy root line with no vector) (n= 6) (p-value= 1.59e-07, one-way ANOVA). Right panel: Relative expression levels of OE-SlEXO1 hairy root line 36 wild type (Hairy root line with no vector) (n= 6) (p-value= 3.12e-05, one-way ANOVA) (Signif. codes: 0 ‘***’ 0.001 ‘**’ 0.01 ‘*’ 0.05). Error bars denote s.d.

**Extended Data Fig. 6:**
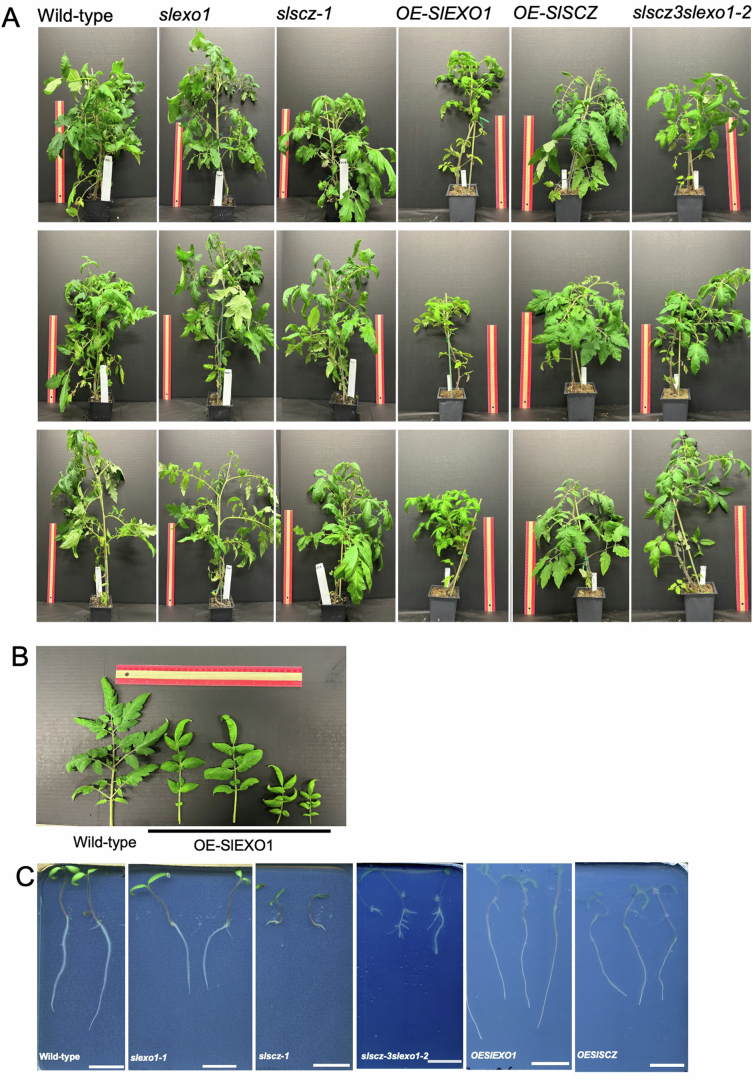
Phenotypes of the aerial part of *slexo1*, *slscz-1, OE-SlEXO1, OE-SlSCZ and slexo1-2slscz-3* plants. **a**. Pictures of 4 weeks old wild-type, *slexo1-1* and *slscz*-1 plants. **b**. Leaves of OE-SlEXO1 are smaller than wild-type leaves. Rule = 30 cm. **c**. Four-day old seedlings of Wild-type, *slexo1-1*, *slscz-1, slscz-3slexo1-2, OE-SlEXO1, OE-SlSCZ*. Scale bar = 2 cm. Ruler = 30cm.

Mining of genes whose transcript abundance is enriched in the endodermis has been successful in identifying regulators of endodermal differentiation^5,33^. We utilized the same approach for the exodermis and focused on transcription factors from multiple families (MYB, NAC, AP2/ERF, MADS-BOX, HD-ZIP, WRKY, bHLH, Zinc finger C2H2 and HOMEOBOX) that are enriched in the exodermis^23^ (Supplementary Fig. 4). Of these, MYB transcription factors were attractive candidates given their known role in endodermal barrier production, secondary cell wall biosynthesis and lignin deposition^15,34–36^. We selected eight MYB family transcription factors for CRISPR/Cas9 mutagenesis and compared lignin deposition in wild type (*R. rhizogenes*-transformed with no binary plasmid) relative to independent mutant alleles of each transcription factor (Supplementary Table 1 and Extended Data Fig. 4). We additionally mutated 13 exodermal-enriched transcription factors (Supplementary Table 1 and Extended Data Fig. 4). A lignin phenotype was only observed for a mutant of a zinc finger C2H2 transcription factor family member, *Solyc09g011120* (Supplementary Fig. 5, Extended Data Fig. 5i–m and Supplementary Table 1, five independent hairy root alleles). Hairy root mutant alleles as well as *A. tumefaciens*-transformed mutant alleles of *Solyc09g011120* (herein *SlEXO1*) had ectopic lignin deposition in the form of a PLC in inner cortex cells in addition to a wild-type exodermal PLC (Fig. 3a,d,e and Extended Data Fig. 5). This phenotype suggests that *SlEXO1* represses PLC formation in an inner cortical layer and thus restricts barrier formation to exodermal cells (Fig. 3d,e). The root length of *slexo1-1* is additionally significantly shorter than that of wild type (Fig. 3c and Extended Data Fig. 6c), but leaf and overall shoot morphology is not affected (Extended Data Fig. 6a,b). Overexpression of *SlEXO1* supports its function as a repressor. In the line with the greatest overexpression, the exodermal PLC and suberization are absent, and with less *SlEXO1* overexpression, there is a decrease in lignin levels with deposition in exodermal cell corners along with occasional epidermal lignification (Fig. 3b,d,e and Extended Data Fig. 5a,n,o). *SlEXO1* overexpression additionally reduces root length (Fig. 3c and Extended Data Fig. 6c), and the shoot and leaves are smaller than the wild type (Extended Data Fig. 6b).

In addition to mining cell type resolution expression data, we selected candidate genes on the basis of their function in root ground tissue patterning and specification in *Arabidopsis*. From these, a heat-shock transcription factor *SCHIZORIZA* regulates asymmetric stem cell divisions and specification of root cortex cell identity^37,38^. In tomato, the cortex–endodermis initial cell gives rise to the endodermis, two inner cortical layers and the exodermis^29^. As the exodermis is the outermost cortex cell layer in tomato, we hypothesized that a tomato homologue of *AtSCZ* could regulate exodermal differentiation. A single putative homologue of *AtSCZ* exists within the tomato genome, *Solyc04g078770* (herein *SlSCZ*) (Supplementary Fig. 6). Two CRISPR/Cas9-edited alleles of *SlSCZ* and a previously published EMS allele, *brt-2* (ref. ^39^), have non-polar lignification (lignin coating all faces of the exodermal cell) in most, but not all exodermal cells (Fig. 3a and Extended Data Fig. 5a–e). When lignin is non-polarized in the exodermal layer, lignin in the inner cortical layer is polarly deposited (Fig. 3a,d,e). Independent hairy root mutant alleles have groups of lignified cells across multiple consecutive inner cortical layers, again with the innermost cell containing polar lignification (Extended Data Fig. 5b–e). While *slscz-1* roots also have a short root (Fig. 3c), the shoot is not affected (Extended Data Fig. 6). Ectopic lignin is also present in the endodermis and the vascular cylinder is asymmetrically organized (Fig. 3a and Extended Data Fig. 5a–e). Overexpression of *SlSCZ* (*OE*-*SlSCZ*) did not suppress exodermal lignification. Instead, the phenotype resembled weak *slscz* mutant alleles as completely lignified exodermal cells and inner cortex cells were identified (Fig. 3a,d,e and Extended Data Fig. 5g,h,s). Different from *slscz* alleles, root patterning was symmetrical (Fig. 3b and Extended Data Fig. 5g,h). The shoot of *OE*-*SlSCZ* showed no obvious morphological phenotypes (Extended Data Fig. 6).

**Extended Data Fig. 7:**
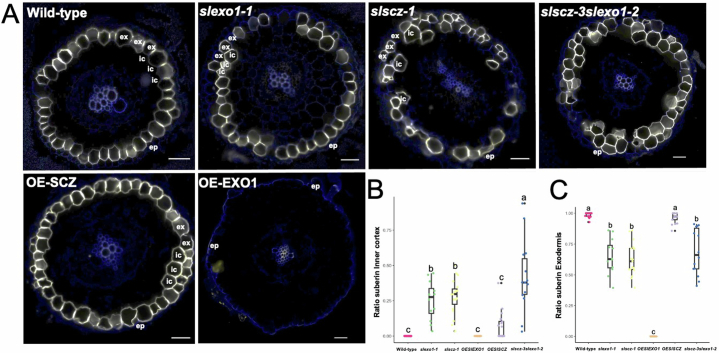
The inner cortex cells in *slexo1-1*, *slscz-1* and *slscz-3slexo1-2* are partially suberized. **a**. The *slexo1-1, slscz-1* and *slscz-3slexo1-2* mutants and OE-SlSCZ have inner cortical layers suberized relative to wild type. The OE-SlEXO1 lacks suberin in both the exodermis and inner cortex. Yellow = fluorol-yellow/suberin, Blue = Autofluorescence. ep = Epidermis, ex = Exodermis, ic = Inner cortex. Scale Bar = 50 µm. **b**. Ratio of suberin in inner cortex, in radial cross-section, was calculated as the number of suberized inner cortex layers observed divided by the total number of inner cortex layers in wild type and *slexo1-1, slscz-1 and slscz-3slexo1-2* mutants roots and SlEXO1 and SlSCZ overexpressors. Statistical significance was determined by one-way ANOVA with a post-hoc Tukey HSD test. pvalue<0.05. Box plots show median, IQR (25^th^-75^th^ percentile), and whiskers represent minima and maxima. Error bars s.d**. c**. Ratio of suberin in inner cortex, in radial cross-section, was calculated as the number of suberized inner cortex layers observed divided by the total number of inner cortex layers in wild type and *slexo1-1, slscz-1 and slscz-3slexo1-2* mutants roots and SlEXO1 and SlSCZ overexpressors. Statistical significance was determined by one-way ANOVA with a post-hoc Tukey HSD test; pvalue<0.05. Box plots show median, IQR (25^th^-75^th^ percentile), and whiskers represent minima and maxima. Error bars s.d.

**Extended Data Fig. 8:**
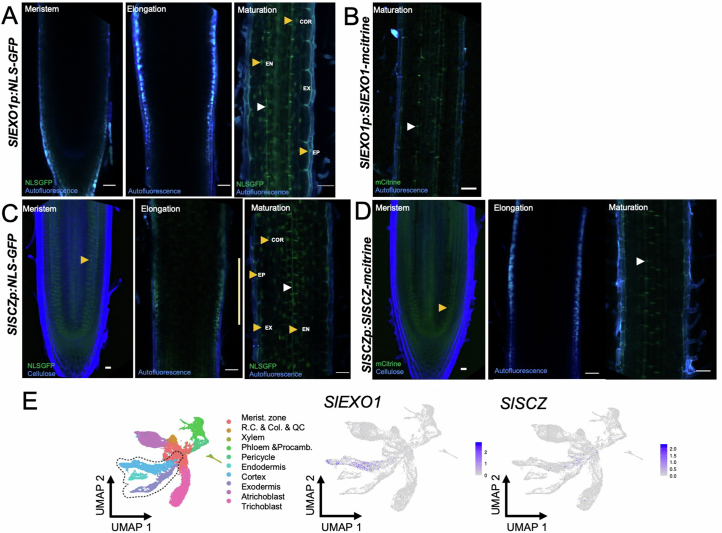
*SlEXO1* and *SlSCZ1* are expressed in cortex and/or exodermis and meristematic region. **a**. *SlEXO1* transcriptional fusion (*SlEXO1*p::*SlEXO1-NLSGFP*). From left to right: Meristem, elongation, and maturation zones. *SlEXO1* is expressed in the epidermis, exodermis, inner cortex, and endodermis in the maturation zone. GFP (Green) and autofluorescence (Blue). Scale Bar = 50 µm. White and orange arrowheads point out the CS autofluorescence and NLS-GFP respectively. **b**. *SlEXO1* promoter fused to the SlEXO1 coding region and mCitrine to show the protein was not detected. White arrowhead points CS. Scale Bar = 50 µm **c**. *SlSCZ* transcriptional fusion (*SlSCZp*::SlSCZ-NLSGFP). From left to right: Meristem, elongation, and maturation. *SlSCZ* is expressed in the endodermis, cortex, and exodermis meristematic cells and the epidermis, exodermis, cortex, and endodermis in the maturation zone. Meristem images: Green= GFP. Blue = Calcofluor White. Elongation and maturation zone images: Green= GFP. Blue = autofluorescence. Scale Bar = 50 µm. White and orange arrowheads point to the CS autofluorescence and NLS-GFP respectively. **d**. *SlSCZ* promoter fused to the *SlSCZ* coding region and mCitrine demonstrates the protein is localized in the stem cell niche, the two inner cortical layers, and the exodermis. The SlSCZ protein was not detected at the elongation or maturation zone. Meristem images: Green= citrine. Blue = Calcofluor White. Elongation and maturation images: Green= citrine. Blue = autofluorescence. Images are captured from *R. rhizogenes* transformed roots. Scale Bar = 50 µm. White and orange arrowheads point to the CS autofluorescence and mCitrine respectively. **e**. Left panel: Annotated single cell clusters from tomato root displayed by an integrated uniform manifold approximation and projection (UMAP). Middle panel: UMAP with *SlEXO1* expression. Right panel: UMAP with *SlSCZ* expression (data from Canto-Pastor et al., 2024).

Given the partial similarities between *slexo1-1* and *slscz-1*, we generated a double mutant to determine their genetic interaction. Multiple double mutant alleles have ectopic (non-polar) lignin in the exodermis, a PLC in the inner cortex and a shorter root (Fig. 3a,d,e and Extended Data Fig. 5p–r). In the exodermis, the symmetric lignin phenotype in *slscz* is partially suppressed by *slexo1* in the double mutant (Fig. 3d). Due to the similarity in the phenotypes for the polar lignin cap in the inner cortex layer, it remains challenging to determine whether these factors are acting additively or epistatically in controlling the exodermis PLC (Fig. 3e). The root length partial recovery in the double mutant demonstrates that *slexo1* partially suppresses the *slscz* short root (Fig. 3c). Exodermis cells are suberized at a later point in root development^23^, and ectopic suberization in inner cortex cells was observed in both single and double mutant alleles of *SlSCZ* and *SlEXO1* (Extended Data Fig. 7a–c), suggesting a change in cell identity of these inner cortex cells. *OE*-*SlSCZ* plants had suberin in inner cortex cells, while there is an absence of suberin in the exodermis and inner cortex in *OE*-*SlEXO1* plants (Extended Data Fig. 7a–c). *SlEXO1* and *SlSCZ* transcripts are enriched in cortex cells, and *SlSCZ* in meristematic cells (Fig. 3f,g and Extended Data Fig. 8e). Expression driven by the *SlEXO1* and the *SlSCZ* promoters fused to nuclear-localized GFP, as well as a translational reporter of SlSCZ (fused to citrine) correlated with the single-cell transcriptome sequencing data (Extended Data Fig. 8a–d).

### *SlSCZ* and *SlEXO1* have partially overlapping downstream regulation

**Fig. 4:**
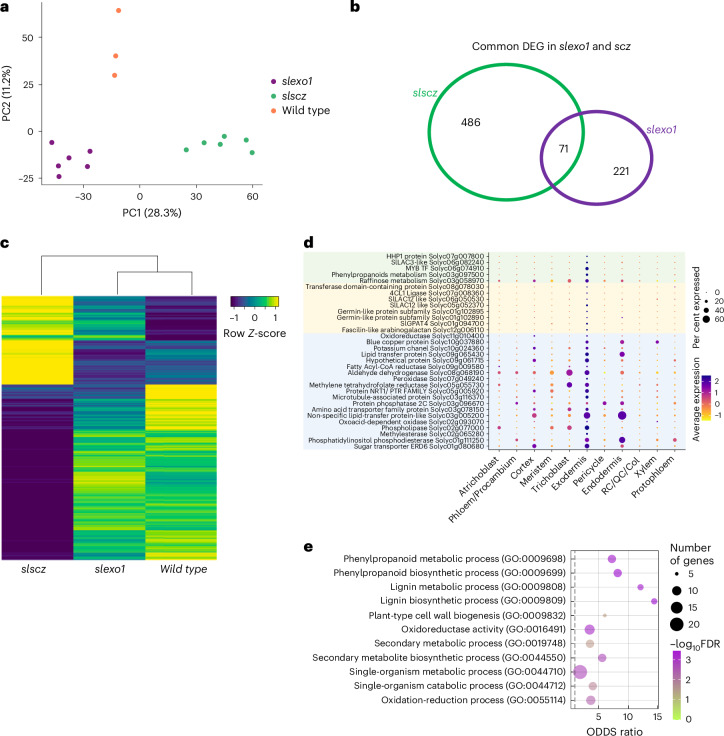
*SlEXO1* and *SlSCZ* transcriptionally regulate distinct and overlapping genes. **a**, PCA of wild type (orange), *slscz* (green) and *slexo1* (purple) of *R. rhizogenes*-transformed root transcriptomes. The first two dimensions contribute to 39.5% of the observed variation. The transcriptomes of these genotypes are distinct in PC1 and PC2. **b**, Venn diagram indicating the common and uniquely DEGs in two independent *slexo1* mutant alleles and two independent *slscz* mutant alleles (FDR = 0.05; fold change ± 1.3) relative to wild type. **c**, Heat map indicating significantly DEGs in each genotype (with *slexo1* and *slscz* representing DEGs in two independent alleles each) relative to wild type. Colour intensity represents the row-normalized *z*-score. Rows are clustered with Pearson correlation and columns with Spearman correlation. **d**, Cell type or tissue-enriched expression profiles for upregulated genes (FDR < 0.05) in *slexo1* and *slscz* mutants. Dot size indicates the percentage of expressing cells and colours represent scaled average expression across developmental stages, with purple colours indicating higher expression levels. Common upregulated genes in *slexo1* and *slscz*, upregulated genes in *slexo1* and upregulated genes in *slscz* are highlighted in green, yellow and blue. **e**, Enriched GO terms for upregulated exodermis-expressed genes in *slexo1* and *slscz-1* (from **d**) relative to wild type.

*SlEXO1* and *SlSCZ* both repress the lignification of inner cortical cell layers, although *SlSCZ* probably does so in a dose-dependent manner given its overexpression phenotype. Their double mutant phenotype further demonstrates that they additively genetically interact (Fig. 3a). Given this genetic interaction and their collective increase in PLC presence and lignification, we conducted transcriptome profiling of two independent alleles each of *slexo1* and *slscz* hairy root mutants to identify their common downstream regulatory pathways. Principal component analysis (PCA) revealed that the transcriptomes of each of these genotypes are transcriptionally distinct (Fig. 4a). *slscz* transcriptomes were more divergent from wild type than *slexo1* (Fig. 4a). Differentially expressed genes (DEGs) were identified in both mutant backgrounds relative to wild type (false discovery rate (FDR) test <0.05) (Fig. 4c), with 71 genes representing common direct or indirect gene targets (Fig. 4b). From the collective upregulated genes, 32 are expressed in the exodermis according to single-cell transcriptome sequencing data^24^ and 5 are commonly upregulated in both mutants (Fig. 4d). These 32 genes are enriched for phenylpropanoid and lignin metabolism or biosynthesis Gene Ontology (GO) categories (FDR test <0.01, Fig. 4e) and are probably involved in the regulation, biosynthesis or polymerization of lignin in the exodermal PLC.

### The exodermal and endodermal barriers are mineral ion checkpoints

**Fig. 5:**
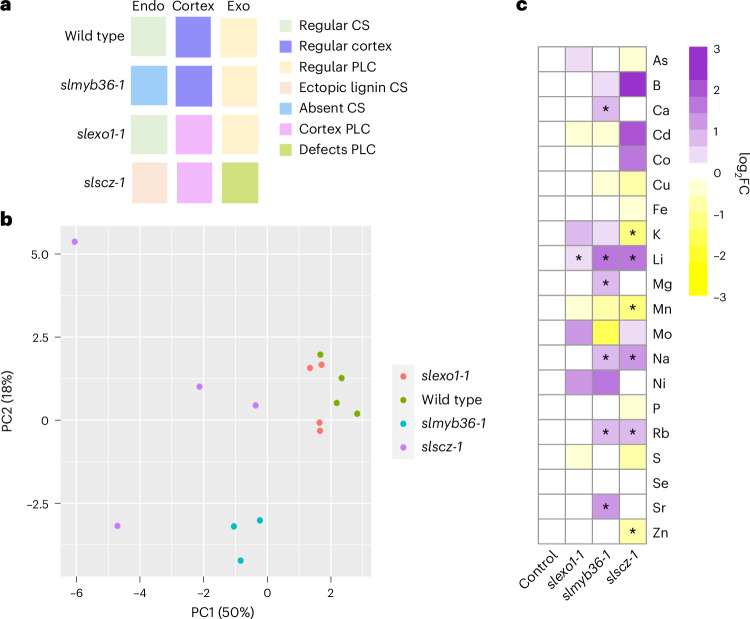
The exodermal PLC barrier does not compensate for the endodermal Casparian strip. **a**, Schematic representation of wild type, *slmyb36-1*, *slexo1-1* and *slscz-1* lignin in the endodermis, cortex and exodermis. **b**, PCA of 20 mineral ions within wild-type, *slmyb36-1, slexo1-1* and *slslscz-1* mutant plants (*n* = 4 for *slscz-1*, *slexo1-1* and wild type; *n* = 3 for *slmyb36-1*). Considerable variation exists between the ionome of the *slscz-1* and *slmyb36-1* mutants. **c**, log_2_ fold change (FC) of ions relative to wild type. Heat map indicates the relative abundance of ions. Statistical significance was determined using one-way ANOVA; *P < 0.05.

**Extended Data Fig. 9:**
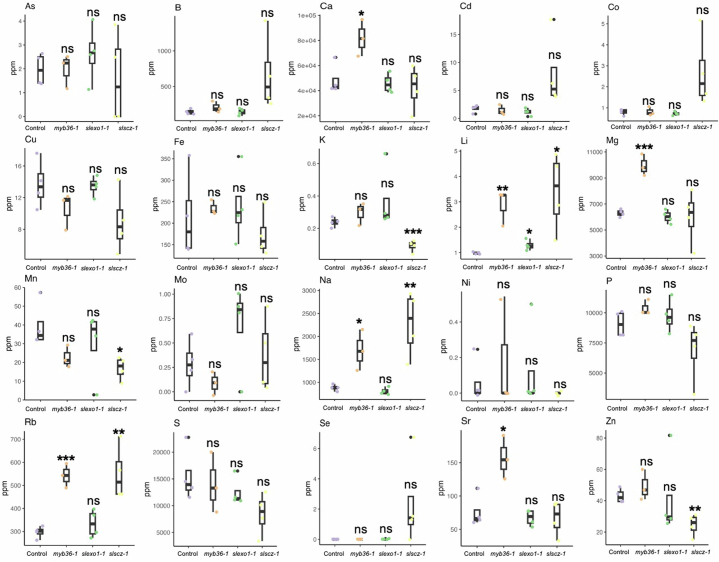
Ion content in *slexo1-1*, *slscz-1*, *slmyb36-*1 and wild-type. Ion content in ppm (Parts per million) for As (Arsenic), B (Boron), Ca (Calcium), Cd (Cadmium), Co (Cobalt), Cu (Cupper), Fe (Iron), K (Potassium), Li (Lithium), Mg (Magnesium), Mn (Manganese), Mo (Molibdenum), Na (Sodium), Ni (Nikel), P (Phosphorous), Rb (Rubidium), S (Sulfur), Se (Selenium), Sr (Strontium), and Zn (Zinc). n=3-4. Statistical significance was determined by one-way ANOVA. Signif. codes: 0 ‘***’ 0.001 ‘**’ 0.01 ‘*’. Box plots show median, IQR (25^th^-75^th^ percentile), and whiskers represent minima and maxima. Error bars s.d.

The endodermal Casparian strip plays an important role in regulating the leaf ionome, consistent with the Casparian Strip as a selective barrier for mineral ion uptake from the cortex to the vasculature and vice-versa^4,15,33,40^. In tomato, both the exodermis and endodermis have apoplastic barriers, leading to the question of whether each barrier has selective control of different ions. We tested this hypothesis by ionome profiling of the tomato shoot in the *slmyb36-1* mutant (CS absence but a wild-type exodermal PLC), the *slexol-1* mutant (no defects in the CS and a PLC in the first inner cortical layer) and the *slscz-1-1* mutant (strong lignification in the exodermis and ectopic lignin in inner cortex cells and the endodermis) (Fig. 5a). Leaves of 4-week-old plants were analysed for their elemental composition using inductively coupled plasma–mass spectrometry (ICP–MS) (Fig. 5b,c). PCA revealed that *slmyb36-1* and *slscz-1* have different ionome profiles than wild type, but the *slexol-1* mutant ionome is more similar to that of wild type (Fig. 5b). Except for lithium, the levels of all ions tested showed no significant differences in *slexo1-1* relative to wild type (one-way analysis of variance (ANOVA), *P* < 0.05). Leaves of *slmyb36-1* accumulate significantly increased sodium (Na), strontium (Sr), rubidium (Rb), calcium (Ca), magnesium (Mg) and lithium (Li) (Fig. 5c and Extended Data Fig. 9). The *slscz-1* ionome accumulated increased levels of sodium (Na), rubidium (Rb) and lithium (Li) and decreased levels of potassium (K), zinc (Zn) and manganese (Mn) (Fig. 5c and Extended Data Fig. 9). Thus, the PLC(s) in *slmyb36-1* does not completely compensate for the absence of the CS, and *slscz-1* with increased lignin displays perturbed leaf ion content, demonstrating that these barriers have unique and overlapping roles in selective mineral ion uptake.

## Discussion

The acquisition of multicellularity required the formation of intercellular barriers to control communication and transport. The best characterized of these in plants is the endodermal CS^1,19,41^. The CS is composed of polymerized lignin that has impregnated the primary cell wall (apoplast) and middle lamellae^1–3,12,19,42–44^. Here we demonstrate that the tomato exodermal barrier is a lignified cell wall that seals the exodermal intercellular and epidermal–exodermal apoplastic space in a polar fashion. Although this structure serves as an apoplastic barrier dependent on its lignin composition, it is not centrally localized, as is the case for CS.

Despite this convergence in function, distinct regulatory mechanisms are responsible for the tomato endodermal CS and the exodermal PLC. Mutant alleles of *SlSHR*, *SlMYB36*, *SlSGN3* and *SlCASP* have defects in endodermal specification and differentiation, but no changes in the PLC. The mostly similar endodermal phenotypes suggest conservation in their function, although there are notable differences. Mutation of tomato *slshr* reduces the number of ground tissue layers and perturbs radial symmetry of the ground tissue and vasculature. In the upper part of the root, a disorganized Casparian strip-like structure and ectopic lignin are observed in an inner cortex layer. These additional phenotypes could reflect expanded and diverse function of *SlSHR* in this species. The *slmyb36* mutant has a complete absence of endodermal lignin (Fig. 2c), while the *atmyb36* mutant has ectopic lignin at endodermal cell corners. The phenotype observed in tomato could only be achieved in *Arabidopsis* when both *AtMYB36* and *AtSGN3* genes were mutated^17^. It is possible that *SlMYB36* controls the expression of *SlSGN3* in tomato. A mutant phenotype was observed when the tomato *slsgn3* was mutated (Fig. 2d), suggesting that a surveillance integrity mechanism does exist in tomato, although it is not necessary to compensate for a lack of the endodermal CS. This could be due to the presence of the PLC as an additional apoplastic barrier rendering this mechanism redundant in tomato. Whether this remains true for other plant species with an exodermis remains to be seen.

**Extended Data Fig. 10:**
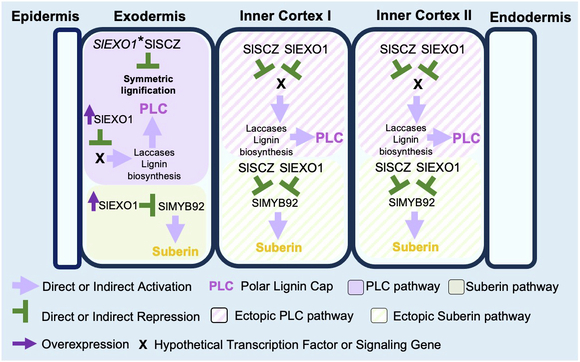
Model for exodermis and inner cortex differentiation. *Exodermis*: SlSCZ genetically interacts with SlEXO1 in repressing symmetric lignification in the PLC pathway. Asterisk = genetic interaction. High levels of ectopic SlEXO1 repress PLC formation and suberization. A hypothetical Transcription factor (X) or Signaling Gene may control the expression of a laccases and/or lignin biosynthesis genes to form the PLC. In a second differentiation step, the transcription factor SlMYB92 (and likely others MYB TFs) activate the suberin biosynthetic pathway (Canto-Pastor et al, 2024)*. Inner Cortex I and II*: SlSCZ and SlEXO1 either directly or indirectly repress X or Signaling Gene which controls PLC formation, suberization, and potentially exodermal specification.

We identified two transcription factors that control polar lignin cap deposition and exodermal cell fate specification. *SlSCZ* regulates the polar deposition of lignin in the exodermis via genetic interaction with *SlEXO1* (Fig. 3a,d). Exodermally expressed genes that are uniquely upregulated in *slscz* are candidates to determine asymmetry in lignin deposition (Fig. 4d). Both *SlSCZ* and *SlEXO1* repress the PLC and probably, exodermal cell fate specification in the inner cortex layer as observed by the presence of the PLC and suberin in the inner cortex layers (Fig. 3a, and Extended Data Figs. 5 and 7). *SlEXO1* is also sufficient to act as a repressor of exodermal lignification and suberization, potentially via *SlMYB92* (ref. ^24^) in a dose-dependent manner (Extended Data Fig. 5), although we were unable to determine whether *SlEXO1* and *SlSCZ* genetically interact. The five exodermally expressed genes that are commonly upregulated in the *slexo1* and *slscz* mutants are candidates for polar lignin cap deposition within the exodermis (Fig. 4d). A proposed model for *SlSCZ* and *SlEXO1* function is presented in Extended Data Fig. 10. Partial suppression of the *slscz* root length phenotype by *slexo1* was also observed (Fig. 3c), although it is unknown how these genes regulate root length. The exodermal defects may reflect their function within the root stem cell niche with a concomitant influence on root length. Alternatively, these two genes may function in distinct developmental contexts.

Genetic perturbation of the endodermal CS alone (*slmyb36-1*) demonstrates that the exodermal PLC cannot fully functionally compensate for the mineral ion barrier function of the CS. Extra lignin in the inner cortical layer of *slexo1-1* did not result in substantial changes in element uptake, showing that in this case, the extra lignin does not act as an additional functional barrier. The symmetric exodermal lignin and inner cortical PLC in the *slscz-1* mutant did function as a barrier perturbing accumulation of unique ions including Zn, Mn and K. A mutant with a complete absence of the exodermal PLC remains to be identified and is necessary to completely delineate the roles of each of these barriers.

In addition to acting as an additional checkpoint for the uptake of mineral nutrients, the exodermal PLC may influence how the root interacts with other abiotic and biotic stimuli. Tolerance of higher ion concentrations in the soil matrix including salinity has been associated with the exodermis^45^ and it may even affect mechanical strength as reported for other root cell types^46^. A breeding advantage may be conferred by the consecutive PLCs of *slexo1-1* in tolerance of higher ion concentrations in the soil matrix or rhizosphere as result of restriction in water availability or high salinity as was reported for a species with a multilayered exodermis^45^. The *Arabidopsis* *scz* mutant is resistant to root knot nematodes^47^ and the same has been speculated for the tomato allele^39^. The tomato exodermal and endodermal barriers could also be selectively modulated for increased symbiosis or resistance to other pathogens^48^.

How common is an exodermal barrier of this shape, composition and function across angiosperms? In addition to our findings in several Solanaceous and a *Solanum* species, an exodermal PLC was described in *Vinca minor*^20^. Barriers of other shapes have been described including a reversed PLC (Y-shape) in *Iris germanica*, and one that resembles a broadened CS in *Trillium grandiflorium*, sugarcane, onion and barley roots^20,45,49^. Plant species with a multiseriate (multilayered) exodermis^21^ resemble these mutant phenotypes. The *AtSCZ* gene regulates cortex cell identity in *Arabidopsis*^37,38^. Given its expression in the meristem, *SlSCZ* may have evolved to regulate cortex cell layer patterning (Fig. 3a). It is intriguing to speculate that *SlSCZ* and *SlEXO1* may enable flexibility in cortex layer number, as well as in restricting exodermal or inner cortical identity, given the suberin deposition in the *slscz* and *slexo1* mutants along with the PLC. These findings have shifted our understanding of the modes by which water and nutrients are taken up and move through the plant root apoplast. The evolutionary origin, underlying molecular determination and function of exodermal barriers dependent on morphology are all exciting questions that remain to be answered. Such flexibility may have influenced how plant roots cope with dynamic and diverse environments and may provide clues as to how to make plants more resilient.

## Methods

### Plant material and growth conditions

*S. lycopersicum* (cv. M82, LA3475) seeds were surface sterilized with 70% ethanol for 5 min and then treated with commercial bleach (70%) for 20 min. Seeds were subsequently washed three times with deionized water. Seeds were transferred to 12 cm × 12 cm plates containing 4.3 g l^−1^ Murashige and Skoog medium (Caisson), 0.5 g l^−1^ MES pH 5.8 and 10 g l^−1^ agar (Difco). Plates were placed in a 23 °C growth chamber for 7–10 days with 16 h of light and 8 h of dark per day. All sections are from 5–6-day-old plants. Alternatively, plants were transferred to soil and placed in a growth chamber at 22 °C with 16/8 h of light/dark conditions. The soil used was Sunshine Mix #1 (Sun Gro Horticulture) irrigated with a complete fertilizer with an N-P-K macronutrient ratio of 2:1:2 in deionized water with no boron.

### Molecular cloning

For the transcriptional fusions, a region of 2–3 kb upstream of the ATG was selected as the promoter region. This PCR fragment was subcloned into D-TOPO entry vector (Fisher Scientific, 450218). The D-TOPO clone containing the promoter fragment was recombined using an LR reaction into the vector pMK7FNFm14GW^50^ using the LR Gateway cloning system (Thermo Fisher).

For the translational fusions, a region of 2–3kb upstream of the ATG was selected as the promoter region. This fragment was cloned in 5 primeTOPO (Thermo Fisher, 591-20), the coding sequence was cloned in D-TOPO (Fisher Scientific, 450218) and the mCitrine cloned in P2P3 Gateway vector (the P2P3 with mCitrine vector was kindly provided by the lab of Niko Geldner). All three vectors were recombined using multisite LR Gateway cloning into the destination vector pB7m34GW^51^. For the SCZ overexpressor, the D-TOPO vector with coding sequence of *SlSCZ* was recombined in pGWB417 Gateway vector. For the SlEXO1 overexpressor, the complementary (c)DNA including the 5′ and 3′ regions was cloned in the D-TOPO vector and recombined in pGWB402 Gateway vector. Primers used are found in Supplementary Table 3.

### *Rhizobium rhizogenes* transformation

‘Hairy root’ transformation with *R. rhizogenes* (ATCC, 15834) was conducted according to ref. ^52^. Cotyledons from 7–10-day-old plants were cut and immersed immediately in a suspension of *R. rhizogenes* containing the desired binary vector. Cotyledons were then floated on sterile Whatman filter paper and co-cultivated in the dark on MS agar plates (1× vitamins, 3% sucrose, 1% agar) for 3 days at 25 °C in the dark without antibiotic selection. Cotyledons were then transferred to a selective plate (MS with 200 mg l^−1^ cefotaxime, 50 mg l^−1^ kanamycin). Thirty independent antibiotic-resistant roots were subcloned for each transgenic line for future analyses with cefotaxime+kanamycin for 2 rounds of subcloning and maintained in media with cefotaxime after that period.

### *Agrobacterium tumefaciens* transformation

The UC Davis Plant Transformation Facility generated transgenics using *A. tumefaciens* and tissue-culture-based protocols.

### Generation of CRISPR/Cas9-edited constructs for the hairy root mutant screen

Target guide RNAs were designed using the CRISPR-PLANT web tool (https://www.genome.arizona.edu/crispr/CRISPRsearch.html). In cases where CRISPR-PLANT did not specify at least three guides with GC content between 40 and 60%, guides were designed with CRISPR-P V2 (http://crispr.hzau.edu.cn/cgi-bin/CRISPR2/CRISPR) using the U6 snoRNA promoter with <3 mismatches within the target gene coding sequence. Genomic sequences (ITAG3.2) were retrieved from Phytozome (https://phytozome-next.jgi.doe.gov/) and gene maps were constructed with SnapGene. Primers for genotyping were designed with Primer-BLAST software (https://www.ncbi.nlm.nih.gov/tools/primer-blast/). Primer specificity was checked against *S. lycopersicum* using blast in Phytozome (Supplementary Table 2). The guide RNA was cloned using a method adapted from ref. ^53^. In summary, oligos containing the sgRNA PAM sequence were phosphorylated and ligated into pYPQ131-3 vectors and recombined into p278 via Gateway cloning. A p278 vector containing all 3 gRNA expression cassettes was then recombined by Gateway cloning into a pMR286 vector containing Cas9 and Kan resistance expression cassettes^54^. The final CRISPR vector was introduced into *R. rhizogenes* (hairy roots) and *A. tumefaciens* (stable lines) to generate transgenics.

### Relative gene expression levels in overexpressor lines

The primers for *SlSCZ* and *SlEXO1* genes were designed using Primer3 (https://primer3.org/) (Supplementary Table 4). An internal control gene and associated primers were selected for *Expressed* gene (Solyc07g025390)^55^. To test for PCR amplification efficiency, we followed the standard dilution curves method for qPCR using the wild-type cDNA sample. All qPCRs were performed using the Luna Universal One-Step RT–qPCR kit (NEB Ref E3005S), VWR PCR plate, 96-well low-profile, full-skirted (Ref 82006-704) and the CFX384 Real-time System (BIO-RAD).

### Histochemistry and imaging

Hairy roots of transcriptional fusions (*SlCASPs, SlSGN3, SlCIF, SlEXO1* and *SlSCZ*) were imaged using a Zeiss LSM700 confocal microscope (water immersion, ×20 objective) with excitation at 488 nm and emission at 493–550 nm for GFP for mCitrine, and excitation at 555 nm and emission at 560–800 nm for autofluorescence. For the SlSCZ:GFP transcriptional fusion and the *SlSCZ*-mCitrine translational fusion images within the meristematic zone, hairy roots were cleared in Clearsee solution for 2 weeks^56^, stained with calcofluor white for 30 min, washed twice and visualized using a Zeiss Observer Z1 fluorescent microscope. The same protocol was used to image the stable lines of the translational fusion constructs (*SlCASP1p*::SlCASP1-mCitrine and *SlCASP2p*::SlCASP2-mCitrine) and the *slcasp1* *slcasp2* stable CRISPR mutant.

For root sections and their staining, 1 cm root segments were embedded in 3% agarose and sectioned using a vibratome (Leica, VT1000 S) to produce 150–200-µM-thick sections. The sections were then cleared and stained in Clearsee solution with basic fuchsin (Fisher Scientific, 632-99-5) for 30 min, followed by two washing steps in Clearsee solution only. As indicated in the figures, in some cases, the basic fuchsin staining was followed by calcofluor white (PhytoTechnology, 4404-43-7) staining for 30 min and then by two washing steps in Clearsee solution. Imaging of cellulose and lignin was performed using a Zeiss LSM700 laser scanning microscope with the ×20 objective. For basic fuchsin: 550–561 nm excitation and 570–650 nm detection were used. Root samples were mounted in ClearSee solution^56^ and scanned and imaged using the Zeiss Observer Z1 (×20 or ×40 objective). The following settings were used: 550–561 nm excitation and 570–650 nm detection for basic fuchsin, and 405 nm excitation and 425–475 nm detection for calcofluor white. For suberin detection with fluorol yellow, root sections were taken 1 cm below the hypocotyl junction and embedded in 3% agarose. Sections were done as previously described. Fluorol yellow staining was done as in ref. ^57^. Confocal laser scanning microscopy was performed on a Zeiss Observer Z1 confocal 698 with the ×20 objective and GFP filter (488 nm excitation, 500–550 nm emission).

### ICP–MS

Seeds from *myb36-1, slexo1-1, slscz-1* and wild-type M82 were germinated in 1% MS+1% sucrose square plates; 6 days after germination, they were transferred to pots in soil. Four plants per genotype were randomized on a tray and watered two times a week with fertilized water for 1 month. The same portion of the compound leaf was collected from each plant and dried in a Falcon tube in a 60 °C oven for 12 h. Dried tissue was homogenized with a mortar and pestle, and the dried powder was weighed for further analyses. The powder was digested in concentrated nitric acid for 3 h at 100 °C. After digestion, nitric acid was evaporated at 80–100 °C. The same volume of 2% nitric acid was added to each sample. The standards for the 20 elements were from Sigma Aldrich (01969-100ML-F, 01932-100ML, 19051-100ML-F, 36379-100ML-F, 30329-100ML-F, 68921-100ML-F, 06335-100ML, 12292-100ML, 30083-100ML-F, 74128-100ML, 68780-100ML, 00462-100ML, 28944-100ML-F, 38338-100ML, 01444-100ML, 18021-100ML, 50002-100ML, 75267-100ML) and Fisher Scientific (CLZN22M, CLFE2-2Y) and were prepared in a dilution series. Samples were analysed in a Perkin Elmer ICP–MS. Calibration curves were made using the standards, and the amount (parts per million) was calculated on the basis of the calibration curve. All elements analysed were detected above the ICP–MS detection limits. Data analyses were performed in R for the PCA plot (prcomp package) and the heat map (pheatmap from Biostrings package). A two-way ANOVA was performed (*P* < 0.05) to identify elements with amounts significantly different from those in wild type. The relative amount for each element per genotype in the heat map was calculated as the log_2_FC from the average of 3–4 replicates.

### Apoplastic tracer assays

Seeds of M82 were grown in 1% MS+1% sucrose. Four days after germination, plants were transferred to new MS square plates with/without 200 µM of piperonylic acid (Sigma, P49805-5G) and 200 µM piperonylic acid plus 10 mM monolignols (Sigma Aldrich, 223735-100MG, 404586-100MG) for 24 h in the dark, with dimethylsulfoxide as the solvent. Segments (3-cm) from the root tip including the root meristem were exposed to 15 µg ml^−1^ PI (P4170, Sigma) in the dark at 30 °C for 1 h. The roots were washed in water and 1-cm segments from the root tip were embedded in 3% agarose and made into sections of 150–200 µM using a vibratome (Leica, VT1000 S). Confocal laser scanning microscopy was performed on a Zeiss LSM700 confocal microscope with the ×20 objective at 405 nm excitation and 600–650 nm detection.

### Transmission electron microscopy

Tomato roots were fixed in 2.5% glutaraldehyde solution (EMS) in phosphate buffer (PB 0.1 M, pH 7.4) for 1 h at room temperature and subsequently fixed in a fresh mixture of 1% osmium tetroxide (EMS) with 1.5% potassium ferrocyanide (Sigma) in PB buffer for 1 h at room temperature. The samples were then washed twice in distilled water and dehydrated in acetone solution (Sigma) in a concentration gradient (30% for 40 min, 50% for 40 min, 70% for 40 min and 100% for 1 h 3 times). This was followed by infiltration in LR White resin (EMS) in a concentration gradient (33% LR White in acetone for 6 h, 66% LR White in acetone for 6 h and 100% LR White for 12 h two times) and finally polymerized for 48 h at 60 °C in an oven in atmospheric nitrogen. Ultrathin sections (50 nm) were cut transversely at 2, 5 and 8 mm from the root tip, the middle of the root and 1 mm below the hypocotyl–root junction using a Leica Ultracut UC7 (Leica), picked up on a 2 × 1mm copper slot grid (EMS) and coated with a polystyrene film (Sigma).

Visualization of lignin deposition in the exodermis and the CS was performed using permanganate potassium (KMnO_4_) staining^25^. The sections were post stained using 1% KMnO_4_ in H_2_O (Sigma) for 45 min and rinsed several times with H_2_O.

Micrographs and panoramic images were taken with an FEI CM100 (FEI) transmission electron microscope at an acceleration voltage of 80 kV with a TVIPS TemCamF416 digital camera (TVIPS) using the software EM-MENU 4.0 (TVIPS). Panoramic images were aligned using the software IMOD^58^.

### Transcriptome profiling and data analysis

For the RNA-seq analyses, 3 cm of the same developmental stage of hairy roots from *slscz-hr5*, *slscz-hr12*, *slexo1-hr6*, *slexo1-hr7* and control hairy roots (wild-type M82 transformed with *R. rhizogenes* with no vector) in three biological replicates. RNA was extracted with the Direct-zol RNA MiniPrep Plus kit (Neta, RPI-ZR2053). cDNA libraries were made with the QuantSeq 3′ mRNA-Seq Library Prep kit from Lexogen (015 QuantSeq FWD 3′ mRNA-Seq Library Prep kit, with single indexing). Sequences were pooled, trimmed and filtered using Trimmomatic^59^. Trimmed reads were pseudo-aligned to the ITAG4.1 transcriptome (cDNA) (Tomato Genome Consortium, 2012) using Kallisto (v.0.43.1)^60^, with the parameters ‘-b 100–single -l 200 -s 30’ to obtain count estimates and transcripts per million (TPM) values. DEGs were detected with the limma R package using normalized counts per million values as required by the package^61^. Counts per million values were normalized with the voom function^62^ using TMM normalization. The functions lmfit, contrasts.fit and ebayes were used to fit a linear model and calculate differential gene expression between the different contrasts. Genes with an adjusted *P* value (*P*_adj_) ≤ 0.05 were considered as differentially expressed. The FDR method was used to control the false discovery rate^63^.

### Expression profiles from single-cell transcriptome sequencing data

The expression profiles of select genes in single-cell transcriptome sequencing data from the tomato root were obtained from ref. ^24^. The plots were generated using the Seurat and scCustomize packages in R.

### Phylogenetic tree construction

The following pipeline from ref. ^23^ was utilized. Forty-two representative proteomes were downloaded from Phytozome, Ensembl or consortia sites depending on availability. These include early diverging taxa and broadly representative taxa from angiosperms. Next, blastp was used to identify homologous sequences within each proteome on the basis of a sequence of interest, with options ‘-max_target_seqs 15 -evalue 10E-6 -qcov_hsp_perc 0.5 -outfmt 6’. To refine this set of sequences, a multiple sequence alignment was generated with MAFFT v.7 (option–auto), trimmed with trimal with the setting ‘-gappyout’, and a draft tree was generated with FastTree. A monophyletic subtree containing the relevant sequences of interest was selected and more distantly related sequences were removed from the list of sequences. For the final trees, MAFFT v.7 using the L-INS-i strategy was used to generate a multiple sequence alignment. Next, trimal was used with the ‘-gappyout’ option. To generate a phylogenetic tree using maximum likelihood, RAxML was used with the option ‘-m PROTGAMMAAUTO’ and 100 bootstraps. Finally, bipartitions with bootstrap values <25% were collapsed using TreeCollapserCL4 (http://emmahodcroft.com/TreeCollapseCL.html). The resulting trees were rooted on sequences from the earliest-diverging species represented in the tree.

### Statistics and reproducibility

All statistical analyses were performed in the R environment (v.3.5.3) and Rstudio (v.1.2.5042). For multiple comparisons between genotypes, a one-way ANOVA followed by a Tukey–Kramer post hoc test was conducted. Groups with *P* < 0.05 were considered significantly different. All bar graphs represent mean ± s.d. Boxplots show the median and interquartile range (IQR, 25th–75th percentiles), with whiskers representing minima and maxima. Filled dots represent individual samples. In all cases, individual biological replicates are indicated as *n*. Experiments and representative images were repeated independently at least three times, unless otherwise noted. Individual *P* values for all statistical analyses are provided in Supplementary Table 5.

### Reporting summary

Further information on research design is available in the Nature Portfolio Reporting Summary linked to this article.

## Supplementary information

Supplementary Information

Reporting Summary

Supplementary Table Table of contents for supplementary tables. Table 1 CRISPR guides and mutation indels description. Table 2 Primers for CRISPR genotyping. Table 3 Primers for transcriptional and translational fusions and overexpressors. Table 4 Primers for qPCR. Table 5 Individual *P* values for all statistical analyses in this study.

## Data Availability

RNA-seq data have been deposited at GEO (GSE215074). CRISPR-generated mutant lines are available upon request with costs to cover sterilization and pathology checks for production of a phytosanitary certificate, and country of destination-specific seed import regulations.
