# Supplementary material for: Regulation and function of a polarly localized lignin barrier in the exodermis
Source: Nat Plants. 2024 Dec 2;11(1):118–30. doi: 10.1038/s41477-024-01864-z (PMC11757151; doi:10.1038/s41477-024-01864-z)
Supplement: Supplementary file 1 — Supplementary Information [file 41477_2024_1864_MOESM1_ESM.pdf]

nature plants

Supplementary information https://doi.org/10.1038/s41477-024-01864-z

Regulation and function of a polarly localized lignin barrier in the exodermis

In the format provided by the authors and unedited

SUPPLEMENTARY INFORMATION

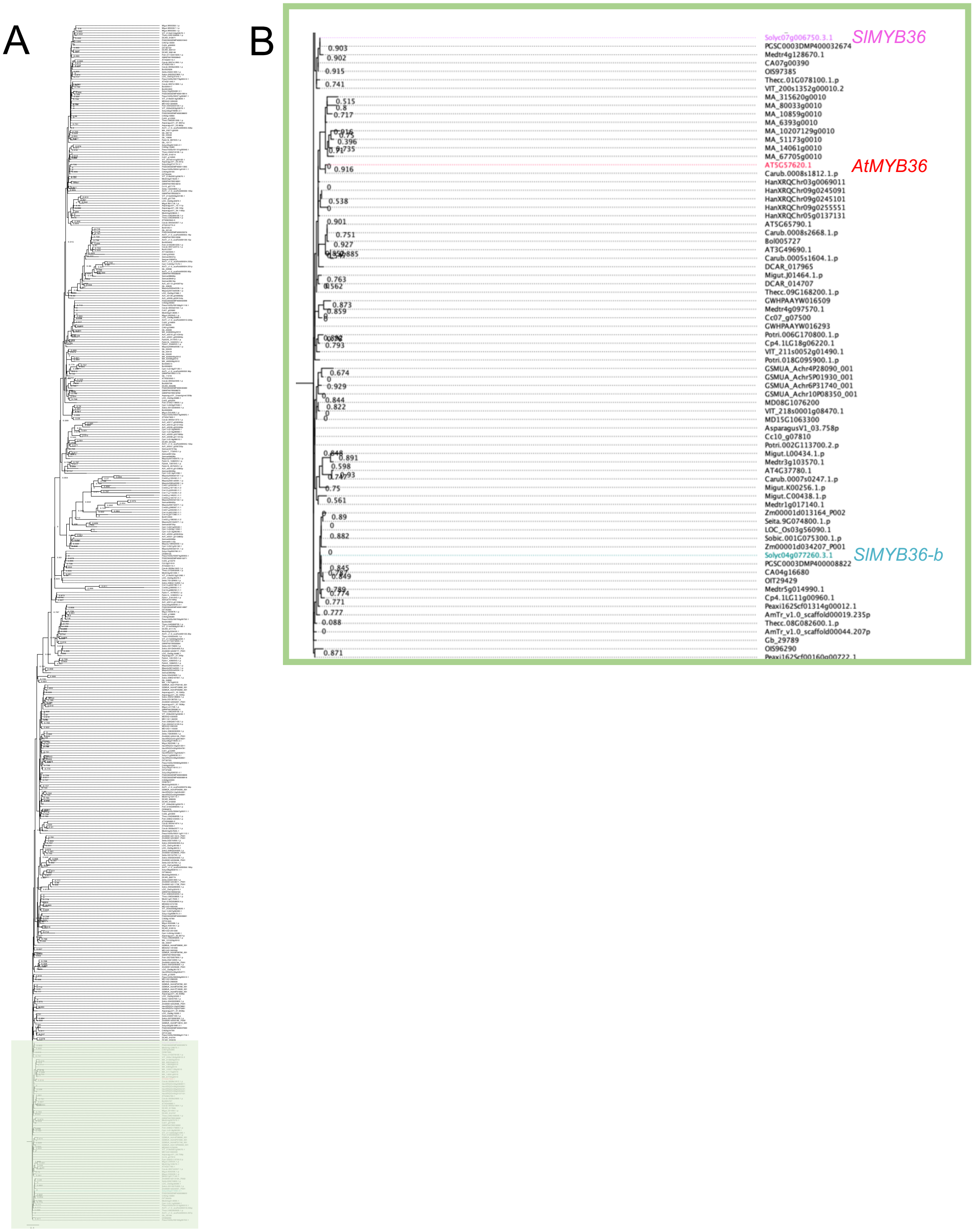

Supplementary Figure 1. ***SlMYB36 phylogeny.* A.** Extended *SlMYB36* phylogeny. Phylogenetic trees generated using protein sequences of several plant species. AmTr: *Amborella trichopoda*, AT: *Arabidopsis thaliana*, Asparagus: *Asparagus officinalis*, Azfi: *Azolla filiculoides*, Bol: *Brassica oleracea*, Carub: *Capsella rubella*, CA: *Capsicum annuum*, Cc: *Coffea canephora*, Cp: *Cucurbita pepo*, DCAR: *Daucus carota*, Gb: *Ginkgo biloba*, HanXRQ: *Helianthus annuus*, MD: *Malus domestica*, Mapoly: *Marchantia*

polymorpha, Medtr: *Medicago truncatula*, Migut: *Mimulus guttatus*, GSMUA: *Musa acuminata*, OIT: *Nicotiana attenuata*, GWHPAAYW: *Nymphaea colorata*, LOC_Os: *Oryza sativa japonica*, Peaxi: *Petunia axillaris*, Pp: *Physcomitrella patens*, MA: *Picea abies*, Potri: *Populus trichocarpa*, Semoe: *Selaginella moellendorffii*, Seita: *Setaria italica*, Solyc: *Solanum lycopersicum*, PGSC: *Solanum tuberosum*, Sobic: *Sorghum bicolor*, Thecc: *Theobroma cacao*, VIT: *Vitis vinifera*, Zm: *Zea mays*. **B.** Detailed *MYB36* phylogeny from green square with *AtMYB36 (At5g57620)* highlighted in red, *SlMYB36* (Solyc07g006750) highlighted in pink, and *SlMYB36-b (Solyc04g077260)* highlighted in blue.

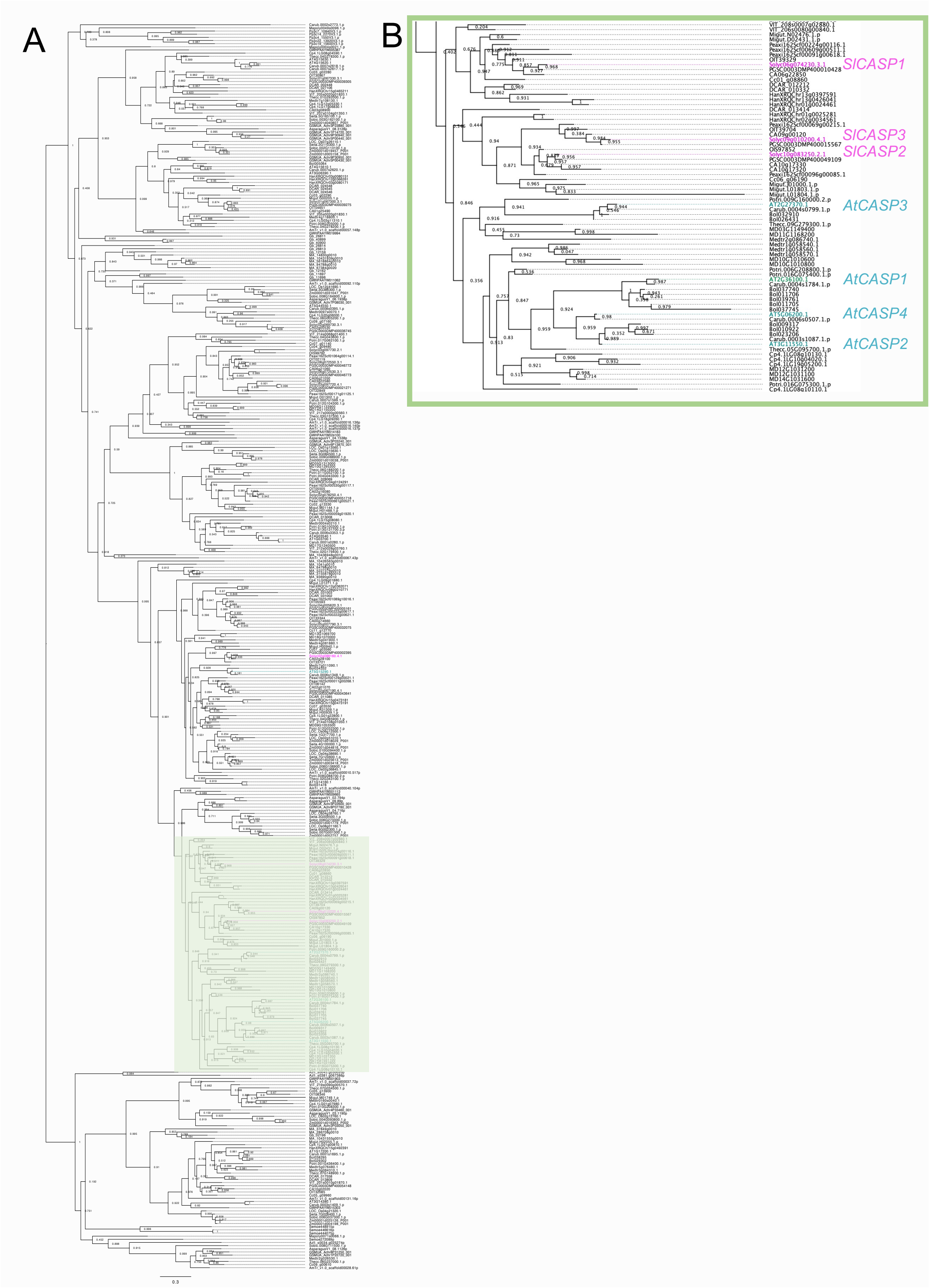

Supplementary Figure 2. *SlCASP* phylogeny. A. Extended *SlCASP* phylogeny. Phylogenetic trees generated using protein sequences of several plant species. AmTr: *Amborella trichopoda*, AT: *Arabidopsis thaliana*, Asparagus: *Asparagus officinalis*, Azfi: *Azolla filiculoides*, Bol: *Brassica oleracea*, Carub: *Capsella rubella*, CA: *Capsicum annuum*, Cc: *Coffea canephora*, Cp: *Cucurbita pepo*, DCAR: *Daucus carota*, Gb: *Ginkgo biloba*, HanXRQ: *Helianthus annuus*, MD: *Malus domestica*, Mapoly: *Marchantia*

polymorpha, Medtr: *Medicago truncatula*, Migut: *Mimulus guttatus*, GSMUA: *Musa acuminata*, OIT: *Nicotiana attenuata*, GWHPAAYW: *Nymphaea colorata*, LOC_Os: *Oryza sativa japonica*, Peaxi: *Petunia axillaris*, Pp: *Physcomitrella patens*, MA: *Picea abies*, Potri: *Populus trichocarpa*, Semoe: *Selaginella moellendorffii*, Seita: *Setaria italica*, Solyc: *Solanum lycopersicum*, PGSC: *Solanum tuberosum*, Sobic: *Sorghum bicolor*, Thecc: *Theobroma cacao*, VIT: *Vitis vinifera*, Zm: *Zea mays*. **B.** Detailed phylogeny from green square in A. *AtCASP* and *SlCASP* genes are colored in blue and pink respectively.

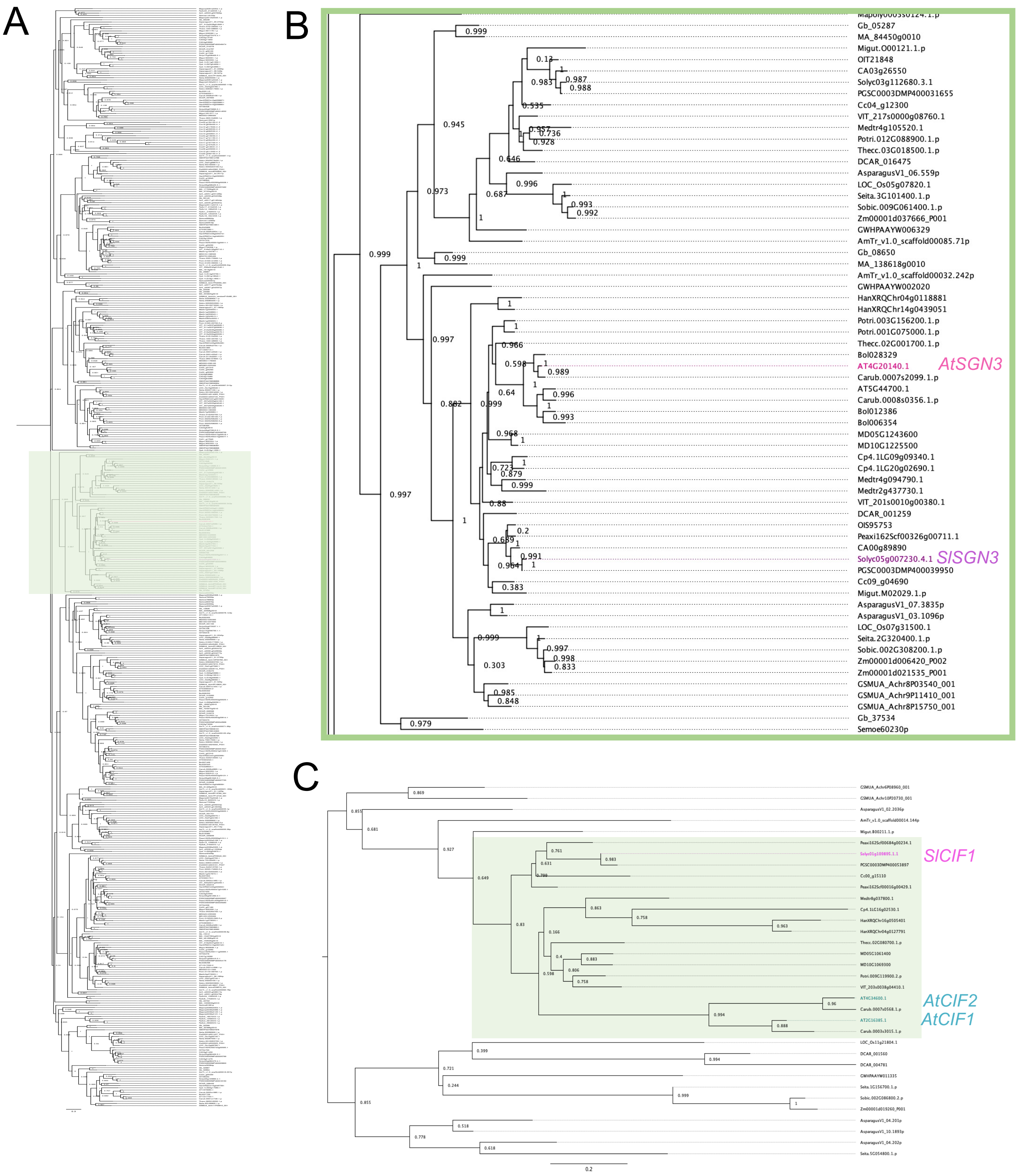

Supplementary Figure 3. *SlSGN3* and *SlCIF1* phylogeny. A. Extended SlSGN3 phylogeny. Phylogenetic trees generated using protein sequences of several plant species. AmTr: *Amborella trichopoda*, AT: *Arabidopsis thaliana*, Asparagus: *Asparagus officinalis*, Azfi: *Azolla filiculoides*, Bol: *Brassica oleracea*, Carub: *Capsella rubella*, CA: *Capsicum annuum*, Cc: *Coffea canephora*, Cp: *Cucurbita*

pepo, DCAR: *Daucus carota*, Gb: *Ginkgo biloba*, HanXRQ: *Helianthus annuus*, MD: *Malus domestica*, Mapoly: *Marchantia polymorpha*, Medtr: *Medicago truncatula*, Migut: *Mimulus guttatus*, GSMUA: *Musa acuminata*, OIT: *Nicotiana attenuata*, GWHPAAYW: *Nymphaea colorata*, LOC_Os: *Oryza sativa japonica*, Peaxi: *Petunia axillaris*, Pp: *Physcomitrella patens*, MA: *Picea abies*, Potri: *Populus trichocarpa*, Semoe: *Selaginella moellendorffii*, Seita: *Setaria italica*, Solyc: *Solanum lycopersicum*, PGSC: *Solanum tuberosum*, Sobic: *Sorghum bicolor*, Thecc: *Theobroma cacao*, VIT: *Vitis vinifera*, Zm: *Zea mays*. **B.** Detailed phylogeny from green square in A. AtSGN3 and SlSGN3 genes are colored in pink and purple respectively. **C.** Extended SlCIF1 phylogeny. Phylogenetic trees generated using protein sequences of several plant species. AmTr: *Amborella trichopoda*, AT: *Arabidopsis thaliana*, Asparagus: *Asparagus officinalis*, Azfi: *Azolla filiculoides*, Bol: *Brassica oleracea*, Carub: *Capsella rubella*, CA: *Capsicum annuum*, Cc: *Coffea canephora*, Cp: *Cucurbita pepo*, DCAR: *Daucus carota*, Gb: *Ginkgo biloba*, HanXRQ: *Helianthus annuus*, MD: *Malus domestica*, Mapoly: *Marchantia polymorpha*, Medtr: *Medicago truncatula*, Migut: *Mimulus guttatus*, GSMUA: *Musa acuminata*, OIT: *Nicotiana attenuata*, GWHPAAYW: *Nymphaea colorata*, LOC_Os: *Oryza sativa japonica*, Peaxi: *Petunia axillaris*, Pp: *Physcomitrella patens*, MA: *Picea abies*, Potri: *Populus trichocarpa*, Semoe: *Selaginella moellendorffii*, Seita: *Setaria italica*, Solyc: *Solanum lycopersicum*, PGSC: *Solanum tuberosum*, Sobic: *Sorghum bicolor*, Thecc: *Theobroma cacao*, VIT: *Vitis vinifera*, Zm: *Zea mays*. *AtCIF1* and *AtCIF2* genes are colored in cyan and *SlCIF1* is colored in magenta.

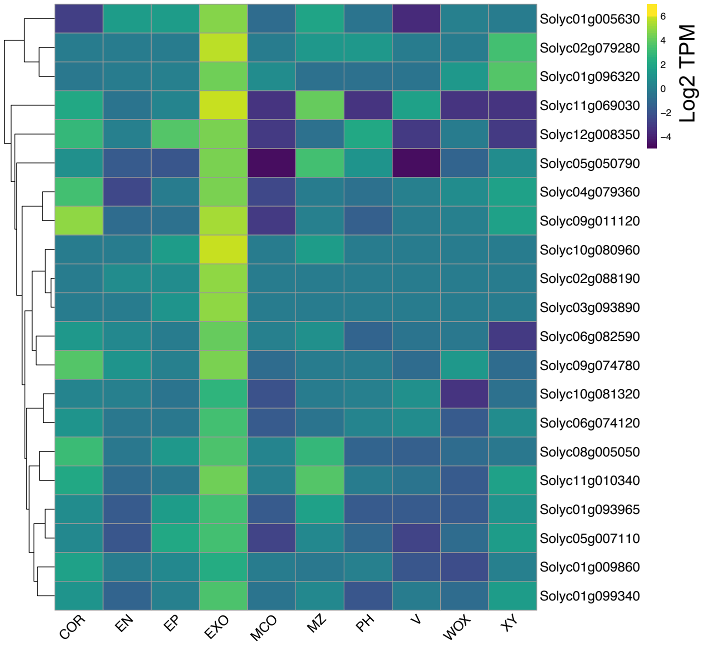

Supplementary Figure 4. The expression of exodermal-enriched transcription factors from Kajala et al., (2021) selected for a hairy root mutant screen. The column names: Epidermis (EP), Exodermis (EXO), Cortex (COR), EN (Endodermis), MCO (Meristematic cortex), Meristematic zone (MZ), Phloem (PH), Vasculature (V), Quiescent center (WOX5), Xylem (XY).

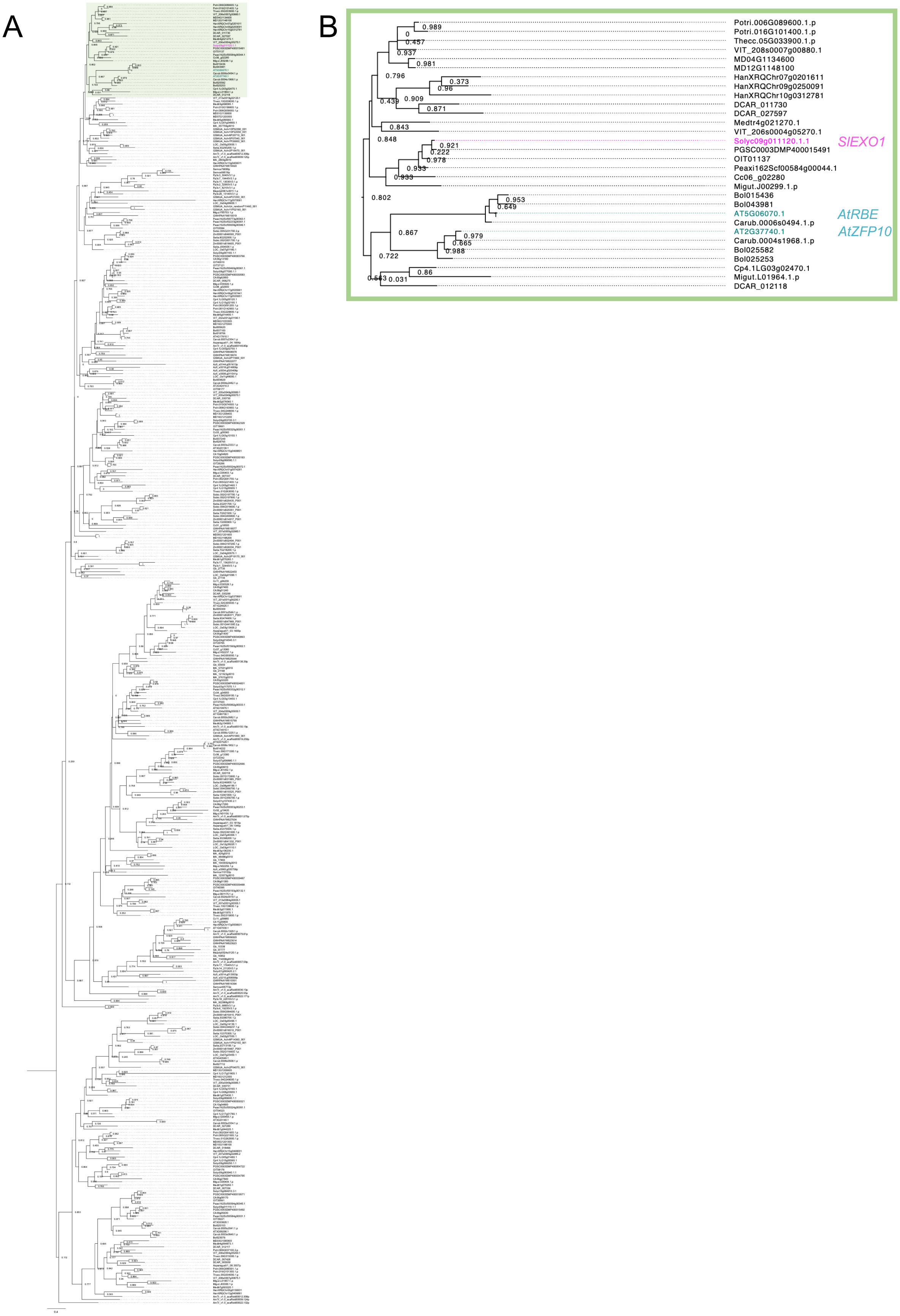
A
B
Potri.006G089600.1.p
Potri.016G101400.1.p
Thecc.05G033900.1.p
VIT_208s0007g00880.1
MD04G1134600
MD12G1148100
HanXRQChr07g0201611
HanXRQChr09g0250091
HanXRQChr10g0312781
DCAR_011730
DCAR_027597
Medtr4g021270.1
VIT_206s0004g05270.1
Solyc09g011120.1.1
SlEXO1
PGSC0003DMP400015491
OIT01137
Peaxi162Scf00584g00044.1
Cc06_g02280
Migut.J00299.1.p
Bol015436
Bol043981
AT5G06070.1
AtRBE
Carub.0006s0494.1.p
AT2G37740.1
AtZFP10
Carub.0004s1968.1.p
Bol025582
Bol025253
Cp4.1LG03g02470.1
Migut.L01964.1.p
DCAR_012118

Supplementary Figure 5. *SlEXO1* phylogeny. A. Extended *SlEXO1* phylogeny. Phylogenetic trees generated using protein sequences of several plant species. AmTr: *Amborella trichopoda*, AT: *Arabidopsis thaliana*, Asparagus: *Asparagus officinalis*, Azfi: *Azolla filiculoides*, Bol: *Brassica oleracea*, Carub: *Capsella rubella*, CA: *Capsicum annuum*, Cc: *Coffea canephora*, Cp: *Cucurbita pepo*, DCAR: *Daucus carota*, Gb: *Ginkgo biloba*, HanXRQ: *Helianthus annuus*, MD: *Malus domestica*, Mapoly: *Marchantia polymorpha*, Medtr: *Medicago truncatula*, Migut: *Mimulus guttatus*, GSMUA: *Musa acuminata*, OIT: *Nicotiana attenuata*, GWHPAAYW: *Nymphaea colorata*, LOC_Os: *Oryza sativa japonica*, Peaxi: *Petunia axillaris*, Pp: *Physcomitrella patens*, MA: *Picea abies*, Potri: *Populus trichocarpa*, Semoe: *Selaginella moellendorffii*, Seita: *Setaria italica*, Solyc: *Solanum lycopersicum*, PGSC: *Solanum tuberosum*, Sobic: *Sorghum bicolor*, Thecc: *Theobroma cacao*, VIT: *Vitis vinifera*, Zm: *Zea mays*. **B.** Detailed phylogeny from the green square in A. *SlEXO1* and the closest *A. thaliana* genes are colored in pink and cyan respectively.

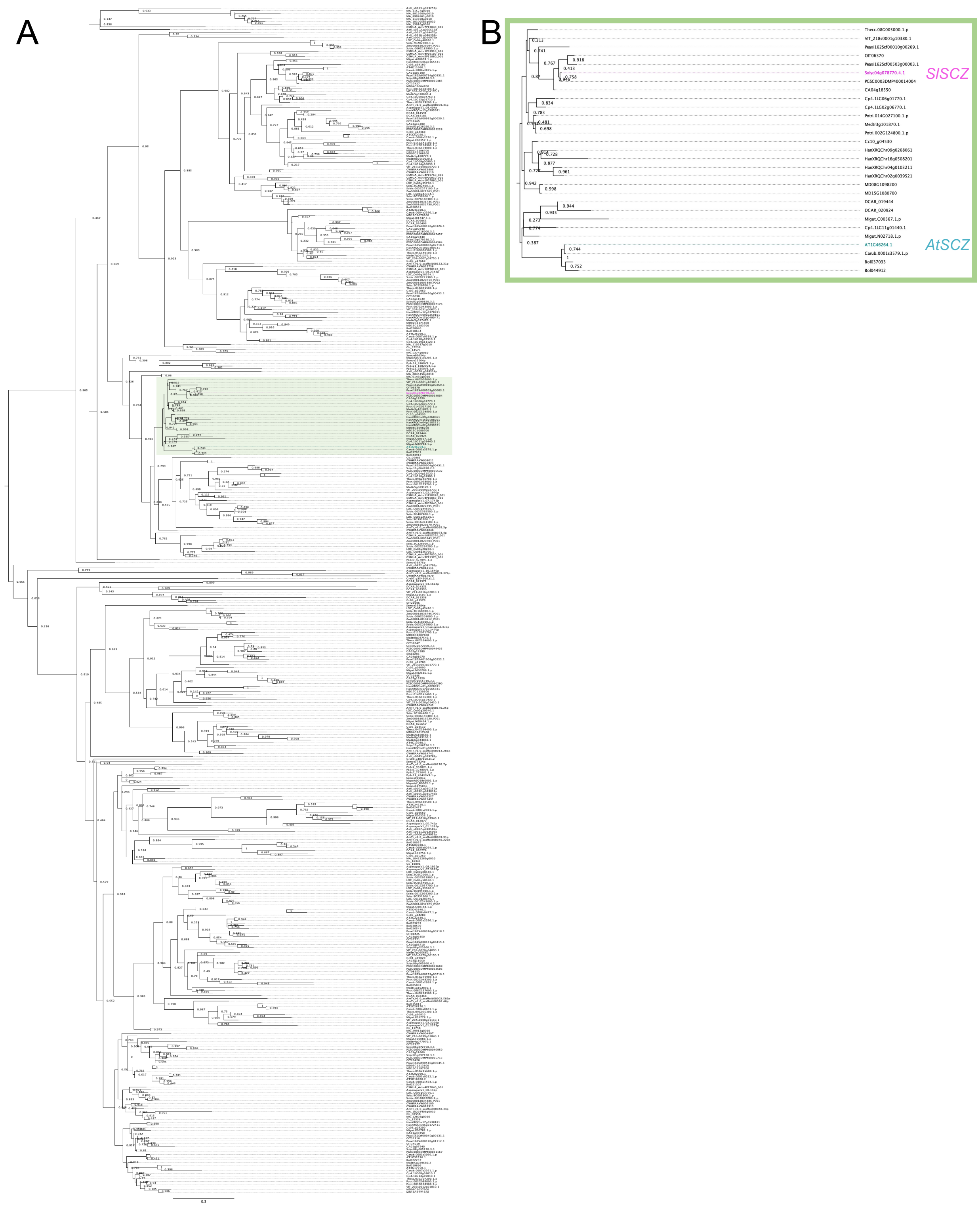

Supplementary Figure 6. *SlSCZ* phylogeny. A. Extended *SlSCZ* phylogeny. Phylogenetic trees generated using protein sequences of several plant species. AmTr: *Amborella trichopoda*, AT: *Arabidopsis thaliana*, Asparagus: *Asparagus officinalis*, Azfi: *Azolla filiculoides*, Bol: *Brassica oleracea*, Carub: *Capsella rubella*, CA: *Capsicum annuum*, Cc: *Coffea canephora*, Cp: *Cucurbita pepo*, DCAR: *Daucus carota*, Gb: *Ginkgo biloba*, HanXRQ: *Helianthus annuus*, MD: *Malus domestica*, Mapoly: *Marchantia polymorpha*, Medtr: *Medicago truncatula*, Migut: *Mimulus guttatus*, GSMUA: *Musa acuminata*, OIT: *Nicotiana attenuata*, GWHPAAYW: *Nymphaea colorata*, LOC_Os: *Oryza sativa japonica*, Peaxi: *Petunia axillaris*, Pp: *Physcomitrella patens*, MA: *Picea abies*, Potri: *Populus trichocarpa*, Semoe: *Selaginella moellendorffii*, Seita: *Setaria italica*, Solyc: *Solanum lycopersicum*, PGSC: *Solanum tuberosum*,

Sobic: *Sorghum bicolor*, Thecc: *Theobroma cacao*, VIT: *Vitis vinifera*, Zm: *Zea mays*. **B.** Detailed phylogeny from the green square in A. AtSCZ and SlSCZ genes are colored in cyan and pink respectively.
