# Supplementary material for: Regulation and function of a polarly localized lignin barrier in the exodermis
Source: Nat Plants. 2024 Dec 2;11(1):118–30. doi: 10.1038/s41477-024-01864-z (PMC11757151; doi:10.1038/s41477-024-01864-z)
Supplement: Supplementary file 2 — Reporting Summary [file 41477_2024_1864_MOESM2_ESM.pdf]

nature portfolio

Corresponding author(s): Concepcion Manzano and Siobhan Mary Brady

Last updated by author(s): 2024/10/03

Reporting Summary

Nature Portfolio wishes to improve the reproducibility of the work that we publish. This form provides structure for consistency and transparency in reporting. For further information on Nature Portfolio policies, see our Editorial Policies and the Editorial Policy Checklist.

Statistics

For all statistical analyses, confirm that the following items are present in the figure legend, table legend, main text, or Methods section.

n/a	Confirmed	
☐	☒	The exact sample size (*n*) for each experimental group/condition, given as a discrete number and unit of measurement
☒	☐	A statement on whether measurements were taken from distinct samples or whether the same sample was measured repeatedly
☐	☒	The statistical test(s) used AND whether they are one- or two-sided<br>*Only common tests should be described solely by name; describe more complex techniques in the Methods section.*
☒	☐	A description of all covariates tested
☒	☐	A description of any assumptions or corrections, such as tests of normality and adjustment for multiple comparisons
☐	☒	A full description of the statistical parameters including central tendency (e.g. means) or other basic estimates (e.g. regression coefficient) AND variation (e.g. standard deviation) or associated estimates of uncertainty (e.g. confidence intervals)
☐	☒	For null hypothesis testing, the test statistic (e.g. *F*, *t*, *r*) with confidence intervals, effect sizes, degrees of freedom and *P* value noted<br>*Give P values as exact values whenever suitable.*
☒	☐	For Bayesian analysis, information on the choice of priors and Markov chain Monte Carlo settings
☒	☐	For hierarchical and complex designs, identification of the appropriate level for tests and full reporting of outcomes
☐	☒	Estimates of effect sizes (e.g. Cohen's *d*, Pearson's *r*), indicating how they were calculated

Our web collection on statistics for biologists contains articles on many of the points above.

Software and code

Policy information about availability of computer code

Data collection	No software was used
Data analysis	R version 3.5.3; R studio Version 1.2.5042, limma R package<br>voom function,TMM normalization, lmfit function, contrasts.fit function, and ebayes function.<br>scCustomize R package.

For manuscripts utilizing custom algorithms or software that are central to the research but not yet described in published literature, software must be made available to editors and reviewers. We strongly encourage code deposition in a community repository (e.g. GitHub). See the Nature Portfolio guidelines for submitting code & software for further information.

Data

Policy information about availability of data

All manuscripts must include a data availability statement. This statement should provide the following information, where applicable:

- Accession codes, unique identifiers, or web links for publicly available datasets
- A description of any restrictions on data availability
- For clinical datasets or third party data, please ensure that the statement adheres to our policy

RNA-seq data have been deposited at GEO (GSE215074). CRISPR-generated mutant lines are available upon request with costs to cover sterilization and pathology checks for production of a phytosanitary certificate, and country-of-destination-specific seed import regulations.

Research involving human participants, their data, or biological material

Policy information about studies with human participants or human data. See also policy information about sex, gender (identity/presentation), and sexual orientation and race, ethnicity and racism.

Reporting on sex and gender	NA
Reporting on race, ethnicity, or other socially relevant groupings	NA
Population characteristics	NA
Recruitment	NA
Ethics oversight	NA

Note that full information on the approval of the study protocol must also be provided in the manuscript.

Field-specific reporting

Please select the one below that is the best fit for your research. If you are not sure, read the appropriate sections before making your selection.

☒ Life sciences ☐ Behavioural & social sciences ☐ Ecological, evolutionary & environmental sciences

For a reference copy of the document with all sections, see nature.com/documents/nr-reporting-summary-flat.pdf

Life sciences study design

All studies must disclose on these points even when the disclosure is negative.

Sample size	For each experiment 5-6 individual plants were examined; an n of >4 is desired to ensure an appropriate estimation of within-sample variability. For CRISPR mutant analysis with A. tumefaciens transformed plants, two independent lines were used for each analysis to minimize influence from transposon insertion location. For R. rhizogenes transformed plants; in negative phenotypic cases 1 line was used; in positive phenotypic cases 3-4 lines were examined due to the increased variability in root phenotypes with this type of transformation. The sample sizes for the experiments are specified in the figure legends and text. Each analysis was conducted with a minimum of three biological replicates, all of which yielded comparable results. No formal statistical methods were used to determine sample size. Instead, the sample sizes were selected empirically, based on prior experience, to maximize the chances of achieving reproducible and statistically significant results.
Data exclusions	No data exclusion
Replication	ICP-MS - 3 biological replicates per genotype; root length - 2 replicates per genotype; Root sections - 2 replicates per genotype; RNAseq - 3 replicates per genotype; All replication attempts were successful.
Randomization	Plants and hairy roots grown in plates - the plates with different genotypes were randomized at the growth chamber; ICP-MS - pots with different genotypes were randomized within the tray.
Blinding	RNA sequencing analysis is fully automated, yielding unbiased and quantitative gene expression data. Phenotyping and confocal imaging were conducted independently by multiple researchers, all of whom obtained consistent results. For the ICP-MS experiments, plants were randomly arranged in trays, and a coded identifier was assigned to each plant to prevent knowledge of the sample material. This coding system was maintained until the ICP-MS results were finalized.

Reporting for specific materials, systems and methods

We require information from authors about some types of materials, experimental systems and methods used in many studies. Here, indicate whether each material, system or method listed is relevant to your study. If you are not sure if a list item applies to your research, read the appropriate section before selecting a response.

Materials & experimental systems

n/a	Involved in the study
☒	☐ Antibodies
☒	☐ Eukaryotic cell lines
☒	☐ Palaeontology and archaeology
☒	☐ Animals and other organisms
☒	☐ Clinical data
☒	☐ Dual use research of concern
☐	☒ Plants

Methods

n/a	Involved in the study
☒	☐ ChIP-seq
☒	☐ Flow cytometry
☒	☐ MRI-based neuroimaging

Dual use research of concern

Policy information about dual use research of concern

Hazards

Could the accidental, deliberate or reckless misuse of agents or technologies generated in the work, or the application of information presented in the manuscript, pose a threat to:

No	Yes
☒	☐ Public health
☒	☐ National security
☒	☐ Crops and/or livestock
☒	☐ Ecosystems
☒	☐ Any other significant area

Experiments of concern

Does the work involve any of these experiments of concern:

No	Yes
☒	☐ Demonstrate how to render a vaccine ineffective
☒	☐ Confer resistance to therapeutically useful antibiotics or antiviral agents
☒	☐ Enhance the virulence of a pathogen or render a nonpathogen virulent
☒	☐ Increase transmissibility of a pathogen
☒	☐ Alter the host range of a pathogen
☒	☐ Enable evasion of diagnostic/detection modalities
☒	☐ Enable the weaponization of a biological agent or toxin
☒	☐ Any other potentially harmful combination of experiments and agents

Plants

Seed stocks

All seeds stocks are Solanum lycopersicum (cv. M82, LA3475). All the transgenics were generated with same cultivar. No seeds were collected from field. All were produced at the green house facilities at UCDavis.

Novel plant genotypes

Rhizobium rhizogenes Transformation
“Hairy root” transformation with R. rhizogenes (ATCC 15834) was conducted according to Ron et al, 2013.Cotyledons from seven to ten-day-old plants were cut and immersed immediately in a suspension of R. rhizogenes containing the desired binary vector. Cotyledons were then floated on sterile Whatman filter paper and co-cultivated in the dark on MS agar plates (1X vitamins, 3% sucrose, % agar) for three days at 25C in the dark, without antibiotic selection. Cotyledons were then transferred to a selective plate (MS with cefotaxime, 200 mg/L, 50 mg/L kanamycin). 30 independent antibiotic-resistant roots were subcloned for each transgenic line for future analyses with Cefotaxime + Kanamycin for 2 rounds of subcloning and maintained in media with Cefotaxime after that period.

Agrobacterium tumefaciens Transformation
The UC Davis Plant Transformation Facility generated transgenics using A. tumefaciens and tissue-culture-based protocols. We obtained 10 independent transgenics lines (T0). Plants were genotyped for the transgene presence and the CRISPR edits. Plants presented in this study are T2 generation.

Generation of CRISPR-Cas9 edited constructs
Hairy root mutant screen Target guide RNAs were designed using the CRISPR-PLANT web tool (https://www.genome.arizona.edu/crispr/CRISPRsearch.html). In cases where CRISPR-PLANT did not specify at least three guides with GC content between 40 to 60%,

Authentication

All transgenic lines were genotyped for transgene insertion. For reporter lines expressing GFP, the signal was analyzed using confocal microscopy. More than five lines were assessed among the A. rhizogenes- and A. tumefaciens-transformed lines. For the overexpression lines, more than five lines were assessed among the A. rhizogenes- and A. tumefaciens-transformed lines. qPCR was also performed to validate overexpression levels. For CRISPR lines, more than 30 A. rhizogenes-transformed lines were sequenced using amplicon sequencing, and more than three positively edited lines were phenotyped for lignin content. A. tumefaciens-transformed T0 plants were genotyped using amplicon sequencing. More than three T2 and/or T3 lines (when viability and seed production allowed) were sequenced by amplicon sequencing and phenotyped for lignin. The phenotypes were compared to those obtained from A. rhizogenes-transformed lines.
